# Ultra-Performance Liquid Chromatography–Tandem Mass Spectrometry Multiple Reaction Monitoring-Based Multi-Component Analysis of Bangkeehwangkee-Tang: Method Development, Validation, and Application to Quality Evaluation

**DOI:** 10.3390/ph18101474

**Published:** 2025-09-30

**Authors:** Chang-Seob Seo

**Affiliations:** KM Science Research Division, Korea Institute of Oriental Medicine, Daejeon 34054, Republic of Korea; csseo0914@kiom.re.kr; Tel.: +82-42-868-9361

**Keywords:** UPLC–MS/MS, Bangkeehwangkee-tang, method development, validation, quality evaluation

## Abstract

**Background/Objectives**: Bangkeehwangkee-tang (BHT) is a traditional herbal formula composed of six medicinal herbs: Sinomenii Caulis et Rhizoma, Astragali Radix, Atractylodis Rhizoma Alba, Zingiberis Rhizoma Recens, Zizyphi Fructus, and Glycyrrhizae Radix et Rhizoma. BHT has been widely used for its immunomodulatory and anti-inflammatory effects. This study aimed to develop a reliable analytical method for the simultaneous determination of 22 marker compounds to ensure consistent quality control and to ensure consistent efficacy in both clinical and non-clinical studies of BHT. **Methods**: An ultra-performance liquid chromatography–tandem mass spectrometry (UPLC–MS/MS) method based on multiple reaction monitoring was developed and validated for the simultaneous determination of 22 marker compounds in BHT. The method was evaluated for selectivity, linearity (coefficient of determination, *r*^2^), sensitivity (limit of detection (LOD) and limit of quantification (LOQ)), accuracy (recovery), and precision (relative standard deviation (RSD)) in accordance with guidelines. **Results**: The developed method exhibited excellent selectivity and linearity (*r*^2^ ≥ 0.9913) for all target compounds. The LOD and LOQ ranged from 0.09 μg/L to 326.58 μg/L and 0.28 μg/L to 979.75 μg/L, respectively. The recovery ranged from 90.36% to 113.74%, and precision (RSD) was ≤15%, confirming the method’s reliability. The application of the method to various BHT samples revealed substantial variations in the marker compound contents, particularly for sinomenine, magnoflorine, and glycyrrhizin. **Conclusions**: These findings highlight the necessity for standardized quality control of BHT and demonstrate that the developed UPLC–MS/MS method is a practical and reliable tool for performing quality assessment of traditional herbal formulas.

## 1. Introduction

Bangkeehwangkee-tang (BHT), known as Boiogito in Japan and Fangji Huangqi tang in China, is a traditional herbal formula composed of six medicinal herbs: Sinomenii Caulis et Rhizoma (SCR), Astragali Radix (AR)**,** Atractylodis Rhizoma Alba (ARA), Zingiberis Rhizoma Recens (ZRR), Zizyphi Fructus (ZF), and Glycyrrhizae Radix et Rhizoma (GRR). It has been widely prescribed in East Asian medicine for its therapeutic effects on immune-related disorders, fatigue, and inflammatory diseases [1,2,3]. Among these, *AR* and *SCR* are particularly recognized for their significant contributions to the immune-modulating and anti-inflammatory effects of BHT [4,5,6,7].

*AR* is well documented for its ability to enhance both innate and adaptive immune functions, alleviate fatigue through antioxidative and adaptogenic mechanisms, and suppress inflammation via modulation of NF-κB and MAPK pathways [4,5]. Similarly, *SCR*, rich in the bioactive compound sinomenine, exhibits potent anti-inflammatory and immunosuppressive activities and has long been traditionally prescribed for rheumatic and edematous disorders [6,7].

The constantly increasing interest in evidence-based herbal medicine has rendered the standardization and quality control of multi-component formulas such as BHT more critical than ever before. However, ensuring consistent quality, safety, and efficacy between the different manufacturing batches remains challenging due to the inherent variability of herbal medicines [8,9]. Factors such as the geographical origin, harvest season, processing methods, and storage conditions can significantly affect the chemical profile and content of bioactive constituents, resulting in batch-to-batch variation even when the same prescription is followed [8,9]. To address these challenges, a scientifically rigorous and validated analytical method is required for the simultaneous quantification of multiple marker compounds in complex herbal preparations [8,9,10].

Among the currently available technologies, liquid chromatography–tandem mass spectrometry (or LC–MS/MS) or ultra-performance LC–MS/MS (UPLC–MS/MS), particularly in multiple reaction monitoring (MRM) mode, has gained wide attention for its high sensitivity, specificity, and reproducibility in simultaneously quantifying multiple bioactive constituents within complex herbal matrices [11,12]. Compared with conventional methods such as high-performance liquid chromatography or UPLC coupled with ultraviolet or evaporative light scattering detectors, LC–MS/MS MRM allows for precise detection of target compounds even at trace levels, making it an ideal tool for evaluating chemical consistency in herbal formulations like BHT [12]. Despite the importance of such approaches, the majority of the previously reported analytical studies have focused on the individual herbal components of BHT [13,14,15,16,17,18]. For instance, previous reports have mainly targeted alkaloids in SCR, saponins and flavonoids in AR, sesquiterpenoids in ARA, phenolic compounds in ZRR, triterpenoid saponins and flavonoids in GRR, and triterpenoids in ZF [13,14,15,16,17,18]. While these studies provide valuable insights into individual constituents, they do not reflect the multi-component interactions or overall chemical profile of BHT as a formula. Therefore, such single-herb approaches are insufficient for ensuring the quality, safety, and therapeutic consistency of BHT in clinical practice. Comprehensive analyses of the entire BHT formulation remain scarce [1,3]. Therefore, the development of a robust and validated LC–MS/MS MRM-based method is warranted for the accurate and simultaneous quantification of key bioactive markers in BHT, facilitating its quality control and standardization as an evidence-based herbal medicine. In addition to LC–MS/MS MRM mode, LC–high resolution MS has also been widely applied for herbal analysis due to its high mass accuracy, structural elucidation, and suitability for both targeted and untargeted profiling [19].

In this study, we developed a simultaneous quantitative method for 22 representative marker compounds in BHT using LC–MS/MS MRM mode. The method was systematically validated in accordance with the analytical method validation guidelines of the International Conference on Harmonisation (ICH), the U.S. Food and Drug Administration (FDA), and the Korea Ministry of Food and Drug Safety (MFDS) [20,21,22]. Our findings provide a scientific basis for the quality assessment of BHT and contribute to the broader standardization of traditional herbal formulations.

## 2. Results and Discussion

### 2.1. Selection of Marker Compounds for Quality Evaluation of BHT Using UPLC–MS/MS with MRM Detection

For the reliable chemical quality evaluation of BHT, a traditional herbal formula composed of six medicinal herbs and 22 marker compounds was selected. The selection was based on their reported presence, isolation or identification from the constituent herbs of BHT, as well as their analytical suitability for UPLC–MS/MS [13,14,15,16,17,23,24,25].

The selected marker compounds represent the major phytochemical classes of BHT, including alkaloids, flavonoids, terpenoids, chalcones, and phenolic compounds. Representative compounds include fangchinoline (FAN), magnoflorine (MAG), sinomenine (SIN), and tetrandrine (TET) from SCR [13]; astragaloside IV (AST IV), calycosin (CAL), calycosin-7-*O*-glucoside (ACLG), formononetin (FOR), and ononin (ONO) from AR [14]; atractylenolide I (ATR I), atractylenolide II (ATR II), and atractylenolide III (ATR III) from ARA [15]; 6-gingerol (GIN) from ZRR [16]; glycyrrhizin (GLY), liquiritin (LIQ), liquiritin apioside (LIQA), liquiritigenin (LIQG), isoliquiritin (ILIQ), isoliquiritin apioside (ILIQA), isoliquiritigenin (ILIQG), and ONO from GRR [17,23]; and rutin (RUT), cinnamic acid (CINA) and MAG from ZF [24,25]. Notably, ONO and MAG were found in multiple herbs of BHT, indicating their broad distribution in the formula. These marker compounds were subsequently employed in the UPLC–MS/MS-based simultaneous quantification to assess the chemical quality of BHT.

Previous studies have reported the chemical profiles of each constituent herb of BHT. Isoquinoline alkaloids such as SIN, MAG, and TET were characterized from SCR by LC–MS/MS [13,26], while 25 flavonoids (e.g., CAL and FOR) and triterpenoids (e.g., AST IV) were simultaneously determined in AR by LC–MS/MS [14,27]. ARA was evaluated for sesquiterpenoids including ATR I, ATR II, and ATR III using HPLC [15,28], and ZRR for GIN and shogaols by LC–MS [16,29]. In ZF, various phenylpropanoids (e.g., CINA and ferulic acid) and flavonoids (e.g., (+)-catechin and hesperidin) were analyzed by LC–MS/MS [30], whereas in GRR, flavonoids (e.g., LIQ and LIQA) and saponins (e.g., GLY) were quantified by UPLC and LC–MS/MS [23,31]. While these studies validate representative markers of individual herbs, they largely focus on single components. In contrast, our study simultaneously quantified 22 markers across all six herbs, providing a more comprehensive and reliable approach for BHT quality evaluation.

### 2.2. MRM Conditions for Simultaneous Determination of 22 Marker Compounds

**Figure 1:**
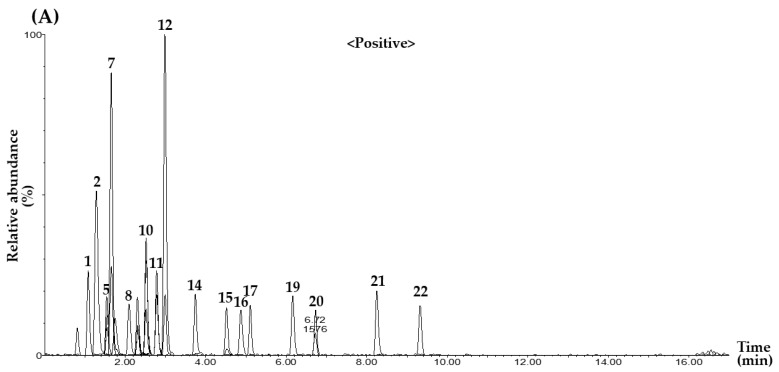

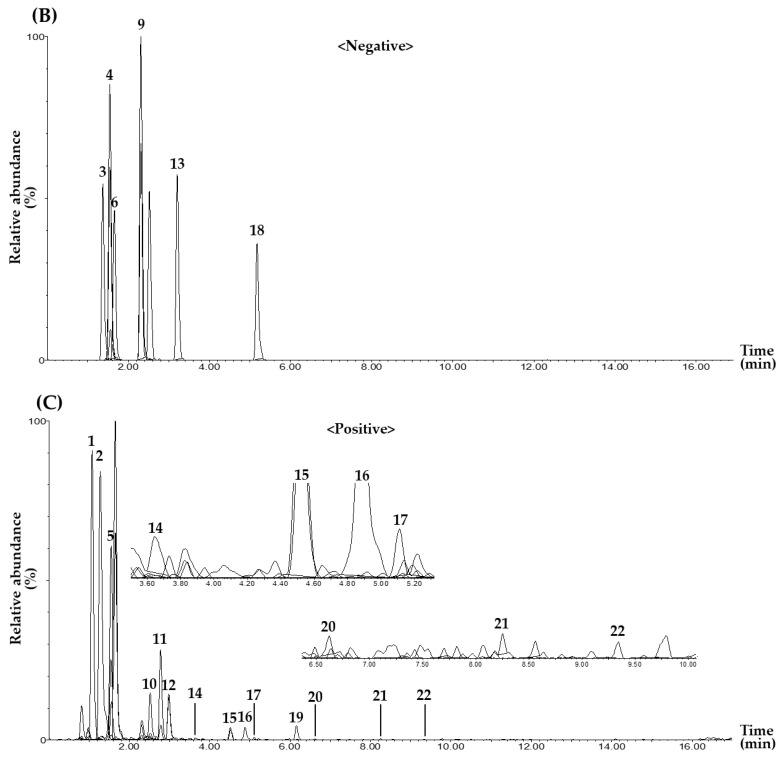

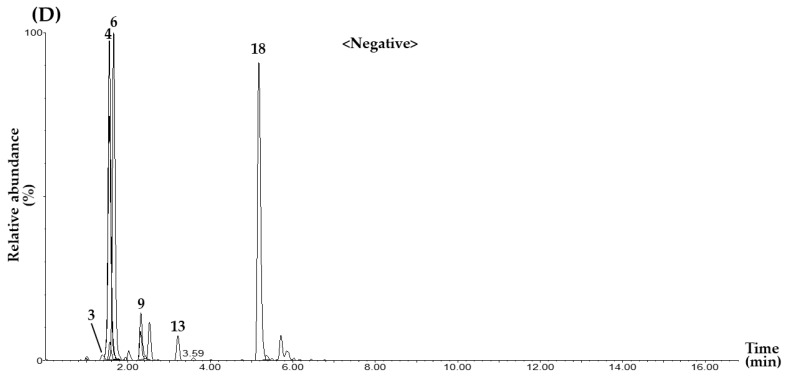
Representative total ion chromatograms of the mixed standard solution (**A**,**B**), and the BHT sample (**C**,**D**) obtained by the UPLC–MS/MS MRM method. The chromatograms were acquired in positive ion mode (**A**,**C**) and negative ion mode (**B**,**D**). The following compounds were detected: SIN (1), MAG (2), RUT (3), LIQA (4), CALG (5), LIQ (6), FAN (7, ND), TET (8, ND), ILIQA (9), ILIQ (10), ONO (11), LIQG (12), CAL (13), CINA (14), ILIQG (15), FOR (16), AST IV (17), GLY (18), GIN (19), ATR III (20), ATR II (21), and ATR I (22). The concentrations of each compound in the mixed standard solution were as follows: 200.00 μg/L (ATR I, FOR, and ILIQG); 250.00 μg/L (ONO), 400.00 μg/L (CAL, CALG, and CINA); 500.00 μg/L (ATR III and LIQG); 800.00 μg/L (AST IV, ATR II, and RUT); 1000.00 μg/L (ILIQA, LIQ, LIQA, and MAG); 1600.00 μg/L (GIN); 2000.00 μg/L (GLY, ILIQ, and SIN); and 4000.00 μg/L (FAN and TET). ND means not detected.

To achieve reliable and simultaneous determination of 22 marker compounds selected from BHT, the UPLC–MS/MS-based MRM analytical conditions were systematically optimized. Specific precursor and product ions were determined for each compound, and various key MS/MS parameters, such as cone voltage and collision energy, were adjusted to ensure high analytical accuracy and sensitivity. The final MRM transitions and optimized parameters for all compounds are summarized in Table 1. The optimized method was successfully applied to both the mixed standard solutions and the BHT samples, and the representative total ion chromatograms are presented in Figure 1. As shown in Figure 1, all 22 marker compounds were efficiently separated and sensitively detected within a single analytical run, confirming the applicability and robustness of the developed method. In addition, the MRM mass spectra, including precursor and product ions, for each of the 22 marker compounds are presented in Figure S1.

### 2.3. Method Validation of the Developed UPLC–MS/MS Assay

The developed UPLC–MS/MS method for the simultaneous determination of 22 marker compounds in BHT samples was validated according to the guidelines of ICH, U.S. FDA, and Korea MFDS [20,21,22]. The validation included selectivity, linearity, sensitivity, accuracy, and precision to demonstrate the method’s reliability for quality evaluation of BHT.

#### 2.3.1. Selectivity

To verify the selectivity, the extracted ion chromatograms of the blank sample, reference standard, and BHT sample of each marker compound were compared (Figure S2). The results showed a clear separation of the target analyte peaks from other matrix components without interference, confirming the selectivity of this method.

#### 2.3.2. Linearity

The linearity of the developed UPLC–MS/MS method was assessed for each of the 22 marker compounds. This was done by evaluating the coefficients of determination (*r*^2^) of calibration curves, which were generated using at least five different concentration levels. As shown in Table 2, all compounds exhibited *r*^2^ ≥ 0.9913, which meets the established criterion of ≥0.99, demonstrating good linearity. These results confirm that the developed method can be reliably applied for the simultaneous determination of all target compounds in BHT samples.

#### 2.3.3. Sensitivity

The LOD and LOQ values for the sensitivity evaluation of the 22 marker compounds ranged from 0.09–326.58 μg/L and 0.28–979.75 μg/L, respectively (Table 2). These results indicate that the developed method provides sufficient sensitivity for the simultaneous determination of all target compounds in BHT samples. Furthermore, the obtained LOD and LOQ values were comparable to or even lower than, those previously reported for major constituents of SCR, AR, ARA, ZF, and GRR analyzed by HPLC or LC–MS/MS [13,14,15,18,23]. These results demonstrate that our method achieves a sensitivity equal to or greater than that of earlier single-herb analyses, while uniquely enabling the simultaneous quantification of 22 structurally diverse marker compounds in a single run.

Taken together, these findings underscore the robustness and practical applicability of the developed UPLC–MS/MS method for reliable quality evaluation of complex herbal formulas.

#### 2.3.4. System Stability

As shown in Table 3, the relative standard deviation (RSD) values for peak areas and retention times from six replicate injections ranged from 0.98–9.87% and 0.08–3.43%, respectively. All values were within the acceptable criteria, confirming the stability of the analytical system.

#### 2.3.5. Accuracy

The recovery of the 20 marker compounds using the developed UPLC–MS/MS MRM method ranged from 90.36% to 111.77%, with the corresponding RSD values being between 0.55% and 11.13% (Table 4). All results met the acceptance criteria of 80–120% across all concentration levels. The accuracy of the method, which was verified through the recovery results, was confirmed to be reliable.

#### 2.3.6. Precision

For the precision evaluation, the intra-day RSD values ranged from 0.89–14.09%, and the inter-day RSD values ranged from 1.09–11.85% (Table 4). Both ranges satisfied the acceptance criterion of ≤15%. These findings confirm that the developed analytical method provides good precision.

### 2.4. Simultaneous Determination of the 22 Marker Compounds in a BHT Sample by the UPLC–MS/MS MRM Method

The newly developed UPLC–MS/MS MRM method was successfully applied to the simultaneous determination of 22 marker compounds in three different BHT samples (BHT–1, BHT–2, and BHT–3). As shown in Table 5, SIN, MAG, and GLY were detected at relatively higher concentrations compared to other marker compounds in all samples, with the highest levels observed in the BHT–1 sample (22.90, 9.42, and 6.44 mg/g, respectively). In contrast, in the BHT–2 and BHT–3 samples, significantly lower levels of these compounds were extracted, particularly BHT–3, which exhibited the lowest concentrations overall. Notably, in the BHT–2 sample, all three major components derived from ARA, namely ATR I, ATR II, and ATR III, were detected at ≤LOQ. Furthermore, FAN and TET were not detected in all samples. These findings indicate that there are considerable differences in the content and composition of marker compounds among the BHT samples, which are likely attributable to variations in the origin and quality of the herbal raw materials, as well as differences in the manufacturing processes. Therefore, this study highlights the necessity of standardization for the quality evaluation of complex traditional herbal preparations or related products, through the application of the simultaneously developed UPLC–MS/MS quantitative analytical method.

## 3. Materials and Methods

### 3.1. Plant Materials

The six raw herbal medicines comprising BHT (Figure S3)—SCR (Menispermaceae, China), AR (Leguminosae, Korea), ARA (Compositae, Korea), ZRR (Zingiberaceae, Korea), ZF (Rhamnaceae, Korea), and GRR (Leguminosae, China)—were obtained from Kwangmyungdang Pharmaceutical (Ulsan, Republic of Korea), a reputable supplier of pharmaceutical-grade herbal materials. Each material had successfully passed the Korea MFDS quality test and subsequently underwent morphological sensory evaluation by Dr. Goya Choi, an herbalist at the Korea Institute of Oriental Medicine (KIOM, Daejeon, Republic of Korea) prior to its use in the study. The voucher specimens of these six raw herbal medicines (Specimen Nos: KE85–1 to KE85–6) have been deposited at KIOM.

### 3.2. Chemicals and Reagents

A total of 22 reference standard compounds for the quality assessment of BHT were purchased from certified suppliers of high-purity natural products such as Wuhan ChemFaces Biochemical (Wuhan, China), PhytoLab GmbH & Co. KG (Vestenbergsgreuth, Germany), Wuhan ChemNorm Biotech Co., Ltd. (Wuhan, China), Shanghai Sunny Biotech Co., Ltd. (Shanghai, China), Chengdu Biopurify Phytochemicals Ltd. (Chengdu, China), Fujifilm Wako Pure Chemical Co. (Osaka, Japan), and Merck KGaA (Darmstadt, Germany). The details of these standard compounds are summarized in Table S1, and chemical structures are presented in Figure S4. LC–MS grade, methanol (>99.9%, CAS No. 67-56-1, catalog No. A456), acetonitrile (100%, CAS No. 75-05-8, catalog No. A955), and formic acid (99.5%, CAS No. 64-18-6, catalog No. A117) were purchased from Thermo Fisher Scientific (Waltham, MA, USA). Ultrapure deionized water with a resistivity of 18.2 MΩ·cm was produced using a Milli-Q Integral 15 water purification system (Merck Millipore, Molsheim, France).

### 3.3. Preparation of BHT Sample

The BHT–1 powder sample was prepared following a previously reported extraction protocol [32]. In brief, 5.0 kg of the six herbal medicines constituting BHT were combined according to the ratios specified in Table S2. The mixed herbal materials were extracted with 50 L of deionized water using an electric extractor (Model No. COSMOS-660, Kyungseo E&P Co. Ltd., Incheon, Republic of Korea) at 100 °C for 2 h. The resulting decoction was filtered through a standard testing sieve (Model No. CG-20341-270, 53 μm mesh, Chunggye Sieve Co., Ltd., Gunpo, Republic of Korea). The obtained filtrate was lyophilized using a freeze-dryer (Model No. PVTFD100R, IlShinBioBase, Dongducheon, Republic of Korea), yielding 911.3 g (18.23%) of BHT–1 powder. The final product was stored at −20 °C under 30% relative humidity until further use. In addition, BHT–2 and BHT–3, which are commercially available BHT products, were purchased from the market and used for analysis.

### 3.4. UPLC–MS/MS Analytical Conditions and Preparation of Standard and Sample Solutions

The UPLC–MS/MS analysis of the BHT samples was conducted using a Waters TQD system (Waters, Milford, MA, USA), with analytical conditions modified from the previously reported methods [32]. Each compound was detected in MRM mode using an Acquity UPLC BEH C18 column (2.1 × 100 mm, 1.7 μm, Waters) maintained at 45 °C. The mobile phase consisted of 0.1% (*v*/*v*) aqueous formic acid and acetonitrile. The detailed analytical conditions and MRM parameters are summarized in Table 1 and Table S3, respectively.

Standard stock solutions were prepared by accurately weighing each reference standard and dissolving it in 70% methanol to obtain a final concentration of 1000 μg/L. The stock solutions were stored in a refrigerator at 4 °C and used for preparing the working standard solutions for the calibration curve by serial dilution. For the preparation of the sample solution for simultaneous determination by UPLC–MS/MS, approximately 50 mg of the sample was accurately weighed into a 10 mL volumetric flask. The volume was made up to the mark with 70% methanol. The mixture was sonicated for 5 min, followed by vortex mixing for 1 min. The solution was filtered through a 0.22-μm hydrophobic polytetrafluoroethylene membrane filter (catalog No. SSKPTFE13022B, SsolKorea, Daejeon, Republic of Korea) and subjected to UPLC–MS/MS analysis by direct injection. For the quantification of SIN, MAG, LIQA, and GLY, the prepared sample solution was further diluted 10-fold prior to analysis.

### 3.5. Validation of the Developed UPLC–MS/MS Method

The validation of the developed analytical method was performed using the BHT–1 sample. This analytical method was systematically evaluated for various key parameters such as selectivity, linearity, sensitivities (LOD and LOQ), accuracy (recovery), and precision in accordance with guidelines of ICH, U.S. FDA, and Korea MFDS [20,21,22]. As a result, the scientific validity and reliability of the method were demonstrated. The evaluation of each parameter was conducted using the following procedure: The selectivity was assessed by confirming that the analyte could be clearly distinguished from other components in the matrix. The linearity was assessed using the *r*^2^ from the regression equation, which describes the correlation between the peak area and the concentration within each compound’s tested range. In accordance with the generally accepted analytical standards, an *r*^2^ ≥ 0.99 was set as the minimum acceptance criterion for linearity. The sensitivity of the method was evaluated by determining the LOD and LOQ based on the signal-to-noise ratios of 3:1 and 10:1, respectively. The system stability of the developed UPLC–MS/MS system was assessed by performing six consecutive replicate injections of the mixed standard solution containing 22 marker compounds. The peak areas and retention times of each compound were measured to assess the stability of signal response (RSD ≤ 10%) and retention time (RSD ≤ 5%). The accuracy was evaluated by performing a recovery test using the standard addition method. Specifically, three different concentrations (low, medium, and high) of the target marker compounds selected for this study were spiked into a known BHT sample. Each BHT sample was individually prepared at a known concentration, followed by sample preparation as described in Section 3.4, and its recovery was subsequently assessed. The acceptance criterion for accuracy was set at 80–120% recovery, based on five replicates (n = 5). The recovery (%) was calculated using the following equation:

$$Recovery\left(\%\right)\;=\;\frac{found\;amount-original\;amount}{spiked\;amount}\times 100$$

The precision was validated by assessing the RSD (%) for intra-day and inter-day measurements. The acceptance criteria were set at ≤15%. Intra-day and inter-day precision were evaluated using five replicates (n = 5) within one day and fifteen replicates (n = 15) over three consecutive days, respectively. The RSD (%) was calculated using the following equation:

$$RSD\left(\%\right)\;=\;\frac{standard\;deviation}{mean}\times 100$$

## 4. Conclusions

In this study, a highly sensitive and reliable UPLC–MS/MS MRM-based analytical method was successfully developed for the simultaneous determination of the 22 marker compounds in BHT. The method was systematically validated in accordance with international guidelines and demonstrated excellent selectivity, linearity (*r*^2^ > 0.99), sensitivity (LOD: 0.09–326.58 μg/L; LOQ: 0.28–979.75 μg/L), accuracy (recovery: 90.36–113.74%), and precision (RSD ≤ 15%). Application of this method to various BHT samples revealed significant differences in the levels of major marker compounds, particularly SIN, MAG, and GLY, underscoring the necessity of standardized quality control for BHT. Importantly, this validated method not only confirms its practical utility for the quality evaluation of traditional herbal formulas but also provides a scientific foundation for its potential adoption into pharmacopoeial standards and for future integration with clinical outcome studies, thereby strengthening the link between chemical consistency and therapeutic efficacy.

## Figures and Tables

**Table 1:** Optimized MRM transitions and UPLC–MS/MS parameters for the simultaneous determination of 22 marker compounds.

Analyte	Ion Mode	Exact Mass (Da)	Precursor Ion (*m*/*z*)	Production Ion (*m*/*z*)	Cone Voltage (V)	Collision Energy (eV)
SIN	Positive	329.16	330.3	181.1	30	35
MAG	Positive	342.17	342.4	297.2	30	20
RUT	Negative	610.15	609.3	300.0	45	30
LIQA	Negative	550.17	549.3	255.0	45	30
CALG	Positive	446.12	447.4	285.2	30	20
LIQ	Negative	418.13	417.4	255.2	30	15
FAN	Positive	608.29	609.5	367.3	30	40
TET	Positive	622.30	623.5	381.0	30	30
ILIQA	Negative	550.17	549.3	255.1	30	30
ILIQ	Positive	418.13	419.3	257.0	35	15
ONO	Positive	430.13	431.3	269.0	25	15
LIQG	Positive	256.07	257.2	137.0	35	25
CAL	Negative	284.07	283.3	268.1	30	20
CINA	Positive	148.05	149.1	131.0	20	10
ILIQG	Positive	256.07	257.2	137.0	15	20
FOR	Positive	268.07	269.1	253.0	40	25
AST IV	Positive	784.46	785.4	143.0	15	20
GLY	Negative	822.40	821.9	351.2	45	40
GIN	Positive	294.18	295.3	177.1	13	10
ATR III	Positive	248.14	249.3	231.2	25	10
ATR II	Positive	232.15	233.3	187.1	35	15
ATR I	Positive	230.13	231.2	185.1	35	20

Sinomenine (SIN), magnoflorine (MAG), rutin (RUT), liquiritin apioside (LIQA), calycosin-7-*O*-glucoside (CALG), liquiritin (LIQ), fangchinoline (FAN), tetrandrine (TET), isoliquiritin apioside (ILIQA), isoliquiritin (ILIQ), ononin (ONO), liquiritigenin (LIQG), calycosin (CAL), cinnamic acid (CINA), isoliquiritigenin (ILIQG), formononetin (FOR), astragaloside IV (AST IV), glycyrrhizin (GLY), 6-gingerol (GIN), atractylenolide III (ATR III), atractylenolide II (ATR II), and atractylenolide I (ATR I).

**Table 2:** Retention times, linear ranges, regression equations, coefficients of determination (*r*^2^), limits of detection (LOD), and limits of quantitation (LOQ) for the 22 marker compounds determined using the developed UPLC–MS/MS MRM method.

Analyte	Retention Time (min)	Linear Range (μg/L)	Regression Equation ^1^ y=ax+b	*r* ^2^	LOD (μg/L)	LOQ (μg/L)
SIN	1.08	1000–16,000	*y* = 3.37*x* − 1383.43	0.9976	6.22	18.65
MAG	1.16	250–4000	*y* = 1.97*x* + 45.57	0.9962	1.35	4.06
RUT	1.16	250–4000	*y* = 1.93*x* + 252.49	0.9922	1.35	4.06
LIQA	1.29	1000–16,000	*y* = 7.45*x* + 7228.74	0.9958	3.16	9.47
CALG	1.39	50–800	*y* = 3.45*x* − 39.60	0.9952	0.47	1.41
LIQ	1.55	250–4000	*y* = 4.77*x* − 609.61	0.9953	22.73	68.20
FAN	1.58	100–1600	*y* = 7.27*x* − 345.76	0.9965	0.91	2.72
TET	1.66	500–8000	*y* = 3.62*x* − 496.01	0.9954	0.54	1.61
ILIQA	1.77	1000–16,000	*y* = 1.00*x* − 1051.84	0.9919	154.58	463.73
ILIQ	2.11	1000–16,000	*y* = 1.22*x* − 1476.09	0.9913	326.58	979.75
ONO	2.32	250–4000	*y* = 5.21*x* − 393.22	0.9950	19.27	57.80
LIQG	2.51	500–8000	*y* = 1.38*x* − 64.59	0.9951	3.81	11.43
CAL	2.80	250–4000	*y* = 23.32*x* − 1153.02	0.9952	0.36	1.08
CINA	3.00	250–4000	*y* = 10.88*x* − 974.76	0.9976	1.26	3.78
ILIQG	3.21	100–1600	*y* = 8.00*x* − 376.07	0.9955	1.14	3.42
FOR	3.75	25–400	*y* = 10.73*x* − 30.91	0.9980	4.99	14.98
AST IV	4.53	25–400	*y* = 17.13*x* − 240.51	0.9954	1.39	4.16
GLY	4.88	50–800	*y* = 14.48*x* + 561.13	0.9973	2.94	8.81
GIN	5.11	100–1600	*y* = 3.83*x* − 150.78	0.9951	0.51	1.53
ATR III	5.17	1000–16,000	*y* = 1.31*x* − 303.46	0.9952	2.45	7.36
ATR II	6.16	100–1600	*y* = 2.47*x* − 130.97	0.9954	2.87	8.60
ATR I	6.72	250–4000	*y* = 9.33*x* − 853.56	0.9953	3.45	10.35

^1^ *y*: peak area of compounds; *x*: concentration (μg/L) of compounds.

**Table 3:** Evaluation of the instrument stability of the developed UPLC–MS/MS system based on retention time and peak area (n = 6).

Analyte	Retention Time (min)			Peak Area		
	Mean	SD ^1^	RSD (%) ^2^	Mean	SD	RSD (%)
SIN	1.08	0.01	1.36	139,394.06	13,122.04	9.41
MAG	1.29	0.01	0.65	125,943.83	9596.52	7.62
RUT	1.39	0.01	0.88	184.97	7.36	3.98
LIQA	1.55	0.02	1.06	41,775.71	2484.52	5.95
CALG	1.58	0.03	1.77	4600.14	311.14	6.76
LIQ	1.66	0.01	0.74	5383.68	299.81	5.57
FAN	1.77	0.06	3.43	2355.67	191.05	8.11
TET	2.11	0.04	1.83	4006.18	188.50	4.71
ILIQA	2.32	0.03	1.41	6746.28	518.66	7.69
ILIQ	2.51	0.02	0.62	264.68	2.58	0.98
ONO	2.80	0.02	0.58	17,323.28	1522.39	8.79
LIQG	3.00	0.01	0.40	2815.17	252.23	8.96
CAL	3.21	0.01	0.23	1391.32	103.60	7.45
CINA	3.75	0.01	0.28	473.03	32.87	6.95
ILIQG	4.53	0.02	0.43	272.82	21.94	8.04
FOR	4.88	0.02	0.42	1514.02	62.55	4.13
AST IV	5.11	0.01	0.16	468.15	43.25	9.24
GLY	5.17	0.00	0.08	18,298.43	1756.04	9.60
GIN	6.16	0.01	0.16	309.83	30.59	9.87
ATR III	6.72	0.01	0.12	2877.97	277.48	9.64
ATR II	8.26	0.01	0.14	461.48	21.65	4.69
ATR I	9.32	0.01	0.10	416.88	33.70	8.08

^1^ SD: standard deviation. ^2^ RSD relative standard deviation.

**Table 4:** Recovery and precision results for the 22 marker compounds using the developed UPLC–MS/MS MRM method.

Analyte	Original Amount (μg/L)	Spiked Amount (μg/L)	Found Amount (μg/L)	Recovery (n = 5)		Precision (RSD, %)	
				Mean (%)	RSD (%)	Intra-Day (n = 5)	Inter-Day (n = 15)
SIN	10,822.20	2000	12,760.32	99.52	2.16	0.89	1.37
		4000	15,116.86	101.99	1.60	3.54	2.02
		8000	19,979.72	106.15	0.96	4.74	2.70
MAG	4771.20	2000	6853.28	101.22	2.14	1.92	2.24
		4000	8771.97	100.01	1.02	3.20	2.22
		8000	13,894.42	108.80	1.66	4.18	2.86
RUT	54.41	100	151.98	98.69	8.38	12.46	8.86
		200	271.16	106.76	6.52	13.82	9.95
		400	474.52	104.52	2.67	9.92	7.55
LIQA	1823.60	500	2319.57	99.85	2.23	2.64	1.88
		1000	2902.25	102.81	3.57	1.40	2.55
		2000	4047.21	105.86	2.22	0.83	2.25
CALG	1386.59	200	1563.87	98.60	1.29	0.92	1.09
		400	1781.05	99.72	3.06	1.70	2.03
		800	2207.05	100.96	1.45	2.68	1.81
LIQ	2378.15	1000	3458.97	102.40	2.35	1.11	2.00
		2000	4516.76	103.17	3.20	2.28	3.15
		4000	6554.79	102.77	1.47	2.22	2.84
ILIQA	2669.20	500	3220.16	101.61	0.55	1.76	1.19
		1000	3786.34	103.20	1.94	3.87	2.57
		2000	4839.43	103.65	2.78	1.57	1.78
ILIQ	4355.81	1000	5207.48	97.25	1.00	3.24	1.82
		2000	6023.68	94.79	2.18	2.43	2.67
		4000	7549.93	90.36	3.37	3.23	3.82
ONO	1519.69	500	2052.02	101.64	1.88	3.64	2.22
		1000	2582.57	102.52	2.15	5.99	3.29
		2000	3616.14	102.76	1.04	1.57	1.85
LIQG	236.40	500	772.83	105.00	3.77	13.16	6.61
		1000	1381.49	111.77	2.91	9.59	5.37
		2000	2351.34	105.16	4.40	5.62	4.59
CAL	254.19	200	480.45	105.83	1.61	8.03	4.73
		400	680.64	104.07	5.53	6.90	5.40
		800	1066.18	101.16	1.77	5.42	3.82
CINA	52.60	50	109.57	107.42	1.94	8.03	6.14
		100	161.03	105.94	3.72	11.25	7.30
		200	255.29	101.31	5.38	7.43	7.28
ILIQG	≤LOQ	50	54.40	108.80	0.56	14.09	7.62
		100	101.62	101.62	10.28	12.28	11.85
		200	200.23	100.12	10.25	8.49	10.82
FOR	145.80	100	233.71	95.39	4.42	7.39	5.47
		200	329.69	95.56	2.54	5.47	4.80
		400	517.32	94.92	3.80	5.27	5.61
AST IV	202.70	200	425.06	105.74	1.93	2.89	2.01
		400	598.90	99.48	9.17	10.55	8.85
		800	1092.25	109.01	2.59	7.89	5.58
GLY	3366.50	2000	5545.27	103.34	3.37	4.34	3.68
		4000	7627.82	103.55	5.65	4.04	4.91
		8000	12,343.69	108.60	2.77	4.24	2.63
GIN	208.97	200	393.50	96.45	6.78	8.22	6.55
		400	619.37	101.87	7.42	6.46	6.76
		800	1075.39	106.69	3.70	8.33	5.76
ATR III	480.20	500	1028.93	104.99	2.76	4.88	3.28
		1000	1556.79	105.19	5.74	11.70	8.50
		2000	2431.67	98.05	6.49	4.81	5.24
ATR II	108.31	100	203.79	97.98	7.07	8.33	7.88
		200	326.53	106.02	3.28	10.56	7.91
		400	495.38	97.51	3.92	6.27	6.33
ATR I	31.60	50	84.92	104.84	3.58	4.09	4.60
		100	132.59	101.22	6.89	13.94	9.12
		200	219.21	94.89	2.38	4.96	3.37

**Table 5:** Simultaneous determination of the 22 marker compounds in BHT samples using the UPLC–MS/MS MRM method (n = 3).

Analyte	BHT–1 ^1^		BHT–2		BHT–3		Source ^2^
	Mean ± SD (mg/g)	RSD (%)	Mean ± SD (mg/g)	RSD (%)	Mean ± SD (mg/g)	RSD (%)	
SIN	22.90 ± 2.16	9.42	2.39 ± 0.06	2.41	1.60 ± 0.16	9.77	SCR
MAG	9.42 ± 0.76	8.01	7.65 ± 0.19	2.52	1.77 ± 0.15	8.60	SCR, ZF
RUT	0.01 ± 0.001	8.31	0.02 ± 0.001	6.00	0.01 ± 0.001	7.75	ZF
LIQA	3.66 ± 0.25	6.95	0.63 ± 0.01	2.03	0.80 ±0.02	1.96	GRR
CALG	0.25 ± 0.02	9.92	0.12 ± 0.003	2.71	0.12 ± 0.01	5.01	AR
LIQ	0.44 ± 0.01	3.23	0.78 ± 0.01	1.64	1.08 ± 0.11	9.88	GRR
FAN	ND ^3^	–	ND	–	ND	–	SCR
TET	ND	–	ND	–	ND	–	SCR
ILIQA	0.47 ± 0.04	8.62	0.09 ± 0.002	2.13	0.11 ± 0.01	5.51	GRR
ILIQ	0.80 ± 0.03	4.31	0.10 ± 0.01	7.46	0.14 ± 0.01	4.46	GRR
ONO	0.26 ± 0.03	9.45	0.09 ± 0.002	2.27	0.10 ± 0.01	6.73	AR, GRR
LIQG	0.04 ± 0.004	9.19	0.10 ± 0.01	8.74	0.09 ± 0.01	8.37	GRR
CAL	0.05 ± 0.002	5.32	0.04 ± 0.004	9.04	0.03 ± 0.001	4.38	AR
CINA	0.01 ± 0.001	6.96	≤LOQ	–	≤LOQ	–	ZF
ILIQG	≤LOQ	–	≤LOQ	–	≤LOQ	–	GRR
FOR	0.03 ± 0.002	7.09	0.02 ± 0.001	4.75	0.01 ± 0.001	9.34	AR
AST IV	0.04 ± 0.004	9.90	≤LOQ	–	0.03 ± 0.002	5.81	AR
GLY	6.44 ± 0.46	7.09	2.33 ± 0.07	3.13	2.86 ± 0.14	5.00	GRR
GIN	0.04 ± 0.03	8.05	0.14 ± 0.01	6.14	0.08 ± 0.01	8.01	ZRR
ATR III	0.08 ± 0.01	9.55	≤LOQ	–	0.12 ± 0.004	3.24	ARA
ATR II	0.02 ± 0.002	9.13	≤LOQ	–	0.08 ± 0.002	2.92	ARA
ATR I	0.01 ± 0.0002	3.64	≤LOQ	–	0.01 ± 0.001	9.14	ARA

^1^ BHT–1: Manufactured by the Korea Institute of Oriental Medicine, BHT–2 and BHT–3: Commercial granules manufactured by pharmaceutical companies in Korea and Japan, respectively. ^2^ Source: SCR: Sinomenii Caulis et Rhizoma, AR: Astragali Radix, ARA: Atractylodis Rhizoma Alba, ZRR: Zingiberis Rhizoma Recens, ZF: Zizyphi Fructus, and GRR: Glycyrrhizae Radix et Rhizoma. ^3^ ND: not detected.

## Data Availability

All data from this study can be found in this paper. Samples of the compounds are available after consultation with the author.

## Supplementary Materials

The following supporting information can be downloaded at: https://www.mdpi.com/article/10.3390/ph18101474/s1, Figure S1: MRM mass spectra of the 22 marker compounds: Precursor ion (Q1, A) and product ion (Q3, B) peaks. Sinomenine (SIN, A), magnoflorine (MAG, B), rutin (RUT, C), liquiritin apioside (LIQA, D), calycosin-7-*O*-glucoside (CALG, E), liquiritin (LIQ, F), fangchinoline (FAN, G), tetrandrine (TET, H), isoliquiritin apioside (ILIQA, I), isoliquiritin (ILIQ, J), ononin (ONO, K), liquiritigenin (LIQG, L), calycosin (CAL, M), cinnamic acid (CINA, N), isoliquiritigenin (ILIQG, O), formononetin (FOR, P), astragaloside IV (AST IV, Q), glycyrrhizin (GLY, R), 6-gingerol (GIN, S), atractylenolide III (ATR III, T), atractylenolide II (ATR II, U), and atractylenolide I (ATR I, V); Figure S2: Extracted ion chromatograms of blanks, standard marker compounds, and BHT samples for selectivity evaluation by UPLC–MS/MS using MRM in positive (A–P) and negative (Q–V) ion modes. SIN (A), MAG (B), CALG (C), FAN (D), TET (E), ILIQ (F), ONO (G), LIQG (H), CINA (I), ILIQG (J), FOR (K), AST IV (L), GIN (M), ATR III (N), ATR II (O), ATR I (P), RUT (Q), LIQA (R), LIQ (S), ILIQA (T), CAL (U), and GLY (V); Figure S3: Six constituent herbal medicines of BHT; Figure S4: Chemical structures of the 22 marker compounds for quality control of BHT; Table S1: Information on the 22 reference standard compounds; Table S2: Composition of the BHT formulation; Table S3: UPLC–MS/MS MRM conditions for simultaneous determination of the 22 marker compounds in the BHT samples.
