# Supplementary material for: Ultra-Performance Liquid Chromatography–Tandem Mass Spectrometry Multiple Reaction Monitoring-Based Multi-Component Analysis of Bangkeehwangkee-Tang: Method Development, Validation, and Application to Quality Evaluation
Source: Pharmaceuticals (Basel). 2025 Sep 30;18(10):1474. doi: 10.3390/ph18101474 (PMC12567129; doi:10.3390/ph18101474)
Supplement: Supplementary file 1 [file pharmaceuticals-18-01474-s001.zip › pharmaceuticals-3868730-supplementary.pdf]

Table S1. Information on the 22 reference standard compounds.

Analyte[1]	Purity (%)	Molecular formula	CAS No.	PubChem CID	Catalog No.	Maker
SIN	99.0	$C_{19}H_{23}NO_4$	115-53-7	5459308	CFN99508	Wuhan ChemFaces Biochemical Co., Ltd. (Wuhan, China)
MAG	98.8	$C_{20}H_{24}NO_4^+$	2141-09-05	73337	CFN98071	Wuhan ChemFaces Biochemical Co., Ltd. (Wuhan, China)
RUT	97.2	$C_{27}H_{30}O_{16}$	153-18-4	5280805	89270	PhytoLab GmbH & Co. KG (Vestenbergsgreuth, Germany)
LIQA	98.8	$C_{26}H_{30}O_{13}$	74639-14-8	10076238	TI042240	Wuhan ChemNorm Biotech Co., Ltd. (Wuhan, China)
CALG	99.4	$C_{22}H_{22}O_{10}$	20633-67-4	71571502	DR10682	Shanghai Sunny Biotech Co., Ltd. (Shanghai, China)
LIQ	99.6	$C_{21}H22O_9$	551-15-5	503737	BP0874	Chengdu Biopurify Phytochemicals Ltd. (Chengdu, China)
FAN	99.0	$C_{37}H_{40}N_2O_6$	436-77-1	73481	BP0582	Chengdu Biopurify Phytochemicals Ltd. (Chengdu, China)
TET	99.7	$C_{38}H_{42}N_2O_6$	518-34-3	73078	BP1379	Chengdu Biopurify Phytochemicals Ltd. (Chengdu, China)
ILIQA	98.8	$C_{26}H_{30}O_{13}$	120926-46-7	6442433	TI042240	Wuhan ChemNorm Biotech Co., Ltd. (Wuhan, China)
ILIQ	98.6	$C_{21}H_{22}O_9$	5041-81-6	5318591	BP0788	Chengdu Biopurify Phytochemicals Ltd. (Chengdu, China)
ONO	98.5	$C_{22}H_{22}O_9$	486-62-4	442813	BP1031	Chengdu Biopurify Phytochemicals Ltd. (Chengdu, China)
LIQG	99.8	$C_{15}H_{12}O_4$	578-86-9	114829	BP0873	Chengdu Biopurify Phytochemicals Ltd. (Chengdu, China)
CAL	99.1	$C_{16}H_{12}O_5$	20575-57-9	5280448	DR10681	Shanghai Sunny Biotech Co., Ltd. (Shanghai, China)
CINA	99.5	$C_9H_8O_2$	140-10-3	444539	035-03412	Fujifilm Wako Pure Chemical Co. (Osaka, Japan)
ILIQG	99.8	$C_{15}H_{12}O_4$	961-29-5	638278	TB02351	Wuhan ChemNorm Biotech Co., Ltd. (Wuhan, China)
FOR	99.1	$C_{16}H_{12}O_4$	485-72-3	5280378	47752	Merck KGaA (Darmstadt, Germany)
AST IV	98.6	$C_{41}H_{68}O_{14}$	84687-43-4	13944297	BP0213	Chengdu Biopurify Phytochemicals Ltd. (Chengdu, China)
GLY	99.1	$C_{42}H_{62}O_{16}$	1405-86-3	14982	BP0682	Chengdu Biopurify Phytochemicals Ltd. (Chengdu, China)
GIN	98.3	$C_{17}H_{26}O_4$	23513-14-6	442793	076-05901	Fujifilm Wako Pure Chemical Co. (Osaka, Japan)
ATR III	98.0	$C_{15}H_{20}O_3$	73030-71-4	155948	DR11040	Shanghai Sunny Biotech Co., Ltd. (Shanghai, China)
ATR II	98.0	$C_{15}H_{20}O_2$	73069-14-4	14448070	DR11039	Shanghai Sunny Biotech Co., Ltd. (Shanghai, China)
ATR I	98.0	$C_{15}H_{18}O_2$	73069-13-3	5321018	DR11038	Shanghai Sunny Biotech Co., Ltd. (Shanghai, China)

[1] Sinomenine (SIN), magnoflorine (MAG), rutin (RUT), liquiritin apioside (LIQA), calycosin-7-*O*-glucoside (CALG), liquiritin (LIQ), fangchinoline (FAN), tetrandrine (TET), isoliquiritin apioside (ILIQA), isoliquiritin (ILIQ), ononin (ONO), liquiritigenin (LIQG), calycosin (CAL), cinnamic acid (CINA), isoliquiritigenin (ILIQG), formononetin (FOR), astragaloside IV (AST IV), glycyrrhizin (GLY), 6-gingerol (GIN), atractylenolide III (ATR III), atractylenolide II (ATR II), and atractylenolide I (ATR I).

Table S2. Composition of BHT formulation.

Herbal name	Scientific name	Family	Used part	Origin	Manufacturing No.	Amount (g)	Ratio (%)
Sinomenii Caulis et Rhizoma	*Sinomenium acutum* (Thunb.) Rehder & E.H.Wilson	Menispermaceae	Rhizome	China	K2631201701	1219.52	24.39
Astragali Radix	*Astragalus mongholicus* Bunge	Leguminosae	Root	Jecheon, Korea	K0491180106	1219.52	24.39
Atractylodis Rhizoma Alba	*Atractylodes japonica* Koidz. ex Kitam.	Compositae	Rhizome	Uljin, Korea	K0902201706	731.70	14.63
Zingiberis Rhizoma Recens	*Zingiber officinale* Rosc.	Zingiberaceae	Rhizome	Seosan, Korea	K2082201701	365.85	7.32
Zizyphi Fructus	*Ziziphus jujuba* Mill.	Rhamnaceae	Fruit	Boeun, Korea	K0702201708	975.61	19.51
Glycyrrhizae Radix et Rhizoma	*Glycyrrhiza uralensis* Fisch.	Leguminosae	Root and rhizome	China	K0011201704	487.80	9.76
					Total	5000.00	100.00

Table S3. UPLC–MS/MS MRM conditions for simultaneous determination of the 22 marker compounds in the BHT samples.

**UPLC conditions**				**MS conditions**	
UPLC system	Acquity UPLC H-Class			MS system	TQD
Column	Acquity UPLC BEH C_{18} column (2.1 mm × 100 mm, 1.7 μm)			MS software	MassLynx v4.2
Column temp.	45 °C			Ion source[1]	ESI^+ and ESI^-
Sample temp.	5 °C			Acquisition mode	MRM
Injection volume	2.0 μL			Capillary voltage	3.0 kV
Flow rate	0.3 mL/min			Cone gas flow	50 L/h
Mobile phase A	0.1% (v/v) formic acid in deionized water			Desolvation gas flow	500 L/h
Mobile phase B	Acetonitrile			Desolvation temp.	300 °C
	Time (min)	A (%)	B (%)	Source temp.	120 °C
Gradient	Initial	80	20		
	0.1	80	20		
	14.0	5	95		
	15.0	0	100		
	15.1	80	20		
	18.0	80	20		

[1] ESI; electrospray ionization, MRM; multiple reaction monitoring

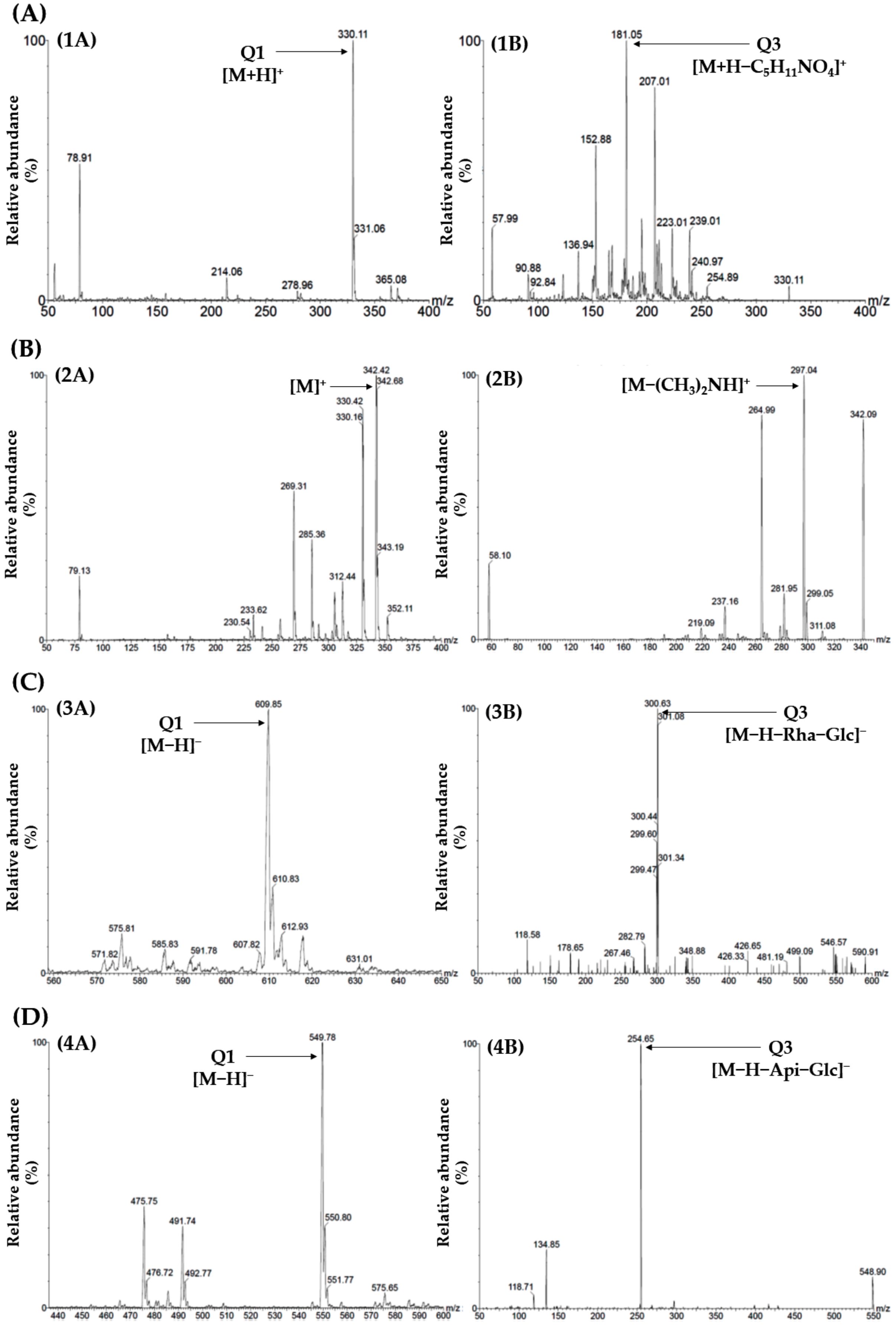

(A)
(1A)
Q1
[M+H]+
330.11
78.91
331.06
214.06
278.96
365.08
(1B)
Q3
[M+H−C5H11NO4]+
181.05
207.01
152.88
57.99
136.94
223.01
239.01
90.88
92.84
240.97
254.89
330.11
(B)
(2A)
[M]+
342.42
342.68
330.42
330.16
269.31
285.36
343.19
79.13
312.44
233.62
230.54
352.11
(2B)
[M−(CH3)2NH]+
297.04
264.99
342.09
58.10
237.16
281.95
299.05
219.09
311.08
(C)
(3A)
Q1
[M−H]−
609.85
610.83
575.81
612.93
585.83
591.78
571.82
607.82
631.01
(3B)
Q3
[M−H−Rha−Glc]−
300.63
301.08
300.44
299.60
301.34
299.47
118.58
178.65
282.79
267.46
348.88
426.65
426.33
481.19
499.09
546.57
590.91
(D)
(4A)
Q1
[M−H]−
549.78
475.75
491.74
550.80
476.72
492.77
551.77
575.65
(4B)
Q3
[M−H−Api−Glc]−
254.65
134.85
118.71
548.90
Relative abundance (%)
m/z

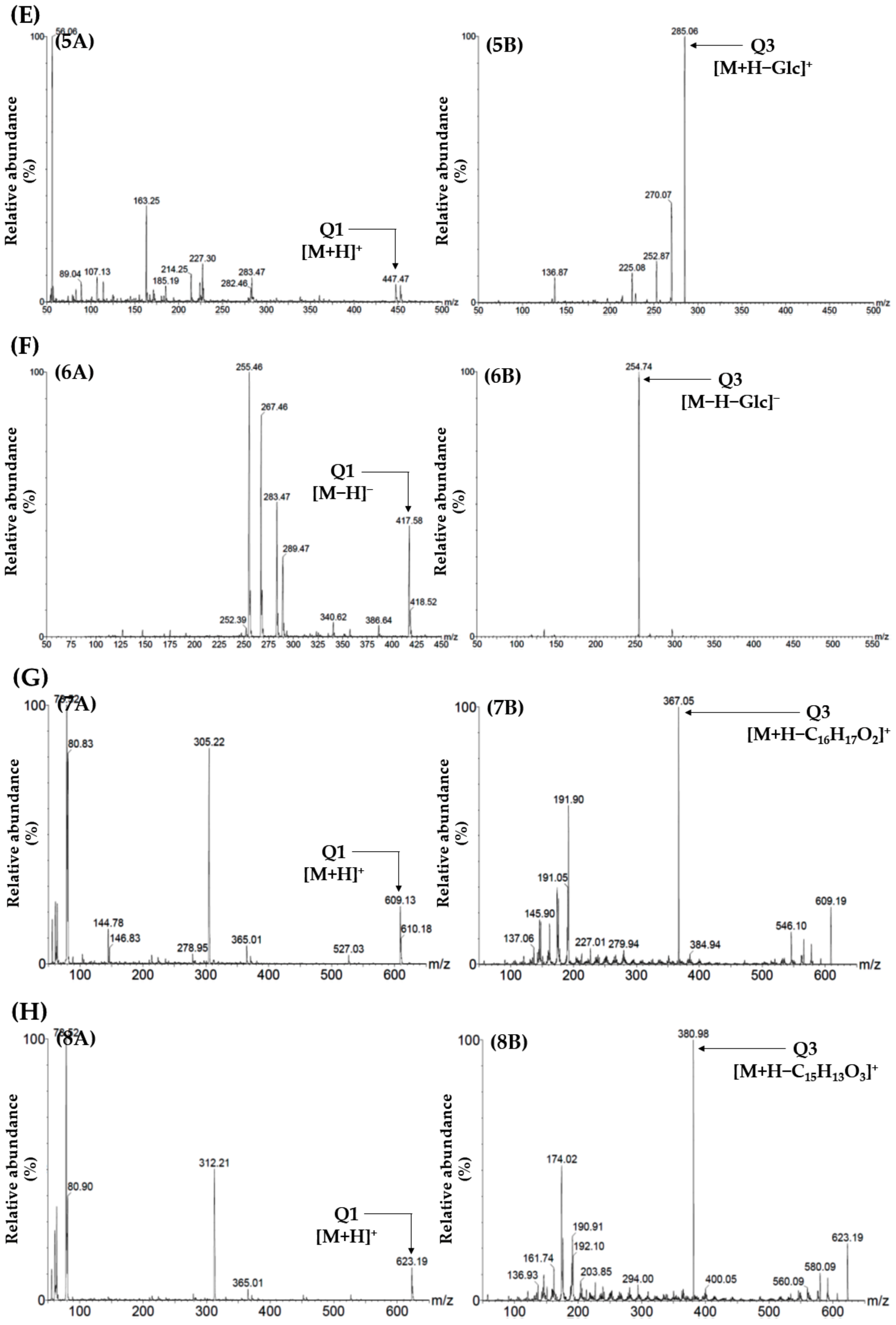

(E)
(5A)
Relative abundance (%)
56.06
163.25
89.04
107.13
185.19
214.25
227.30
282.46
283.47
Q1
[M+H]+
447.47
m/z
(5B)
285.06
Q3
[M+H−Glc]+
270.07
252.87
225.08
136.87
(F)
(6A)
255.46
267.46
283.47
289.47
Q1
[M−H]−
417.58
418.52
252.39
340.62
386.64
(6B)
254.74
Q3
[M−H−Glc]−
(G)
(7A)
80.83
305.22
144.78
146.83
278.95
365.01
527.03
Q1
[M+H]+
609.13
610.18
(7B)
367.05
Q3
[M+H−C16H17O2]+
191.90
191.05
145.90
137.06
227.01
279.94
384.94
546.10
609.19
(H)
(8A)
80.90
312.21
365.01
Q1
[M+H]+
623.19
(8B)
380.98
Q3
[M+H−C15H13O3]+
174.02
190.91
192.10
161.74
136.93
203.85
294.00
400.05
560.09
580.09
623.19

(I)

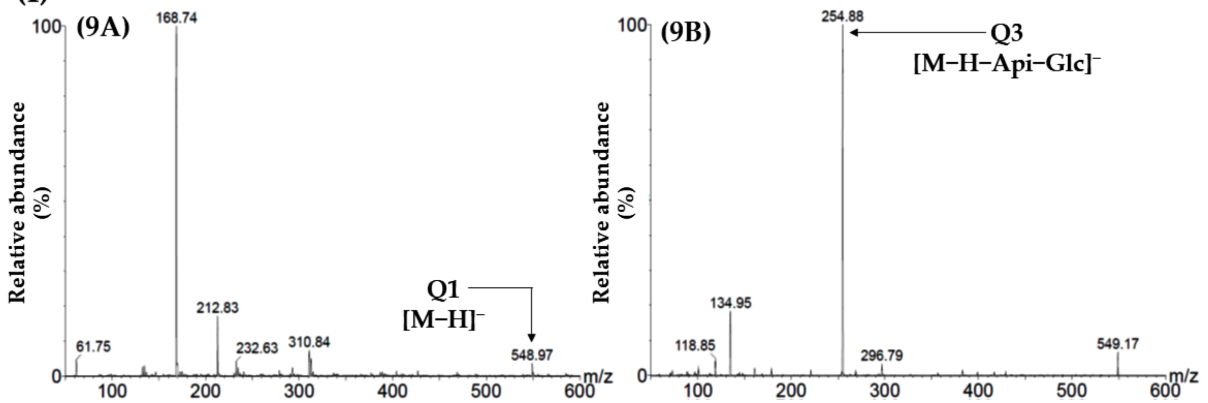

(9A)
100
0
Relative abundance (%)
168.74
212.83
61.75
232.63
310.84
Q1
[M−H]⁻
548.97
m/z
100
200
300
400
500
600
(9B)
254.88
Q3
[M−H−Api−Glc]⁻
134.95
118.85
296.79
549.17

(J)

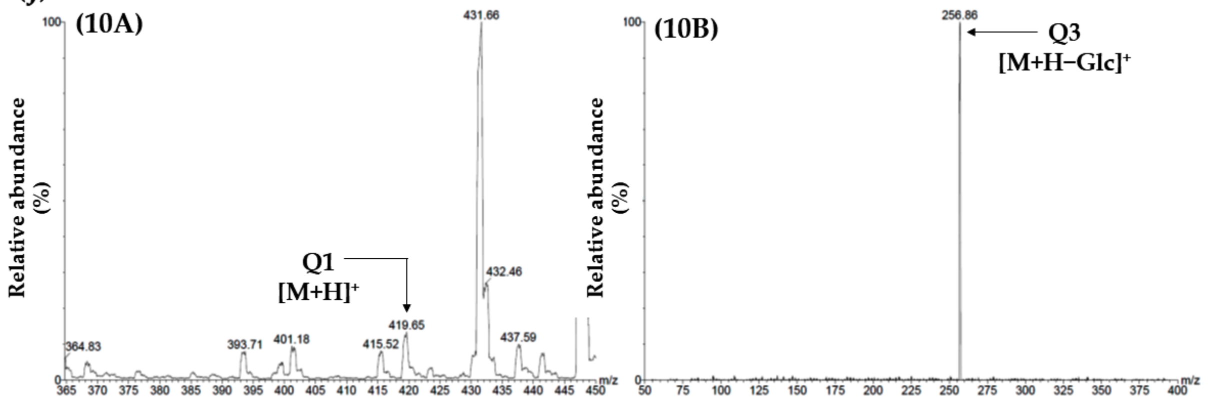

(10A)
Relative abundance (%)
431.66
432.46
Q1
[M+H]⁺
419.65
364.83
393.71
401.18
415.52
437.59
m/z
(10B)
256.86
Q3
[M+H−Glc]⁺

(K)

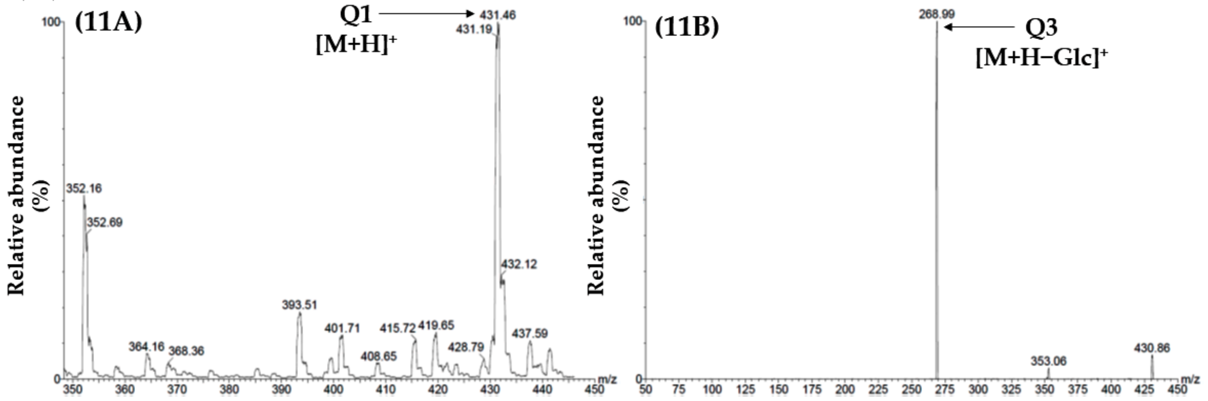

(11A)
Relative abundance (%)
Q1
[M+H]⁺
431.46
431.19
352.16
352.69
432.12
393.51
364.16
368.36
401.71
408.65
415.72
419.65
428.79
437.59
m/z
(11B)
268.99
Q3
[M+H−Glc]⁺
353.06
430.86

(L)

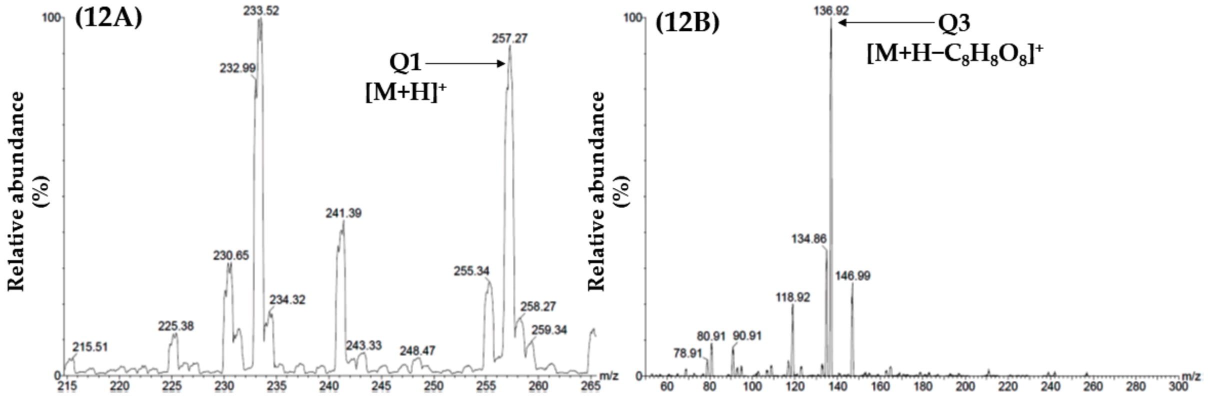

(12A)
Relative abundance (%)
233.52
232.99
Q1
[M+H]⁺
257.27
241.39
230.65
255.34
234.32
258.27
259.34
225.38
215.51
243.33
248.47
m/z
(12B)
136.92
Q3
[M+H−C8H8O8]⁺
134.86
146.99
118.92
80.91
90.91
78.91

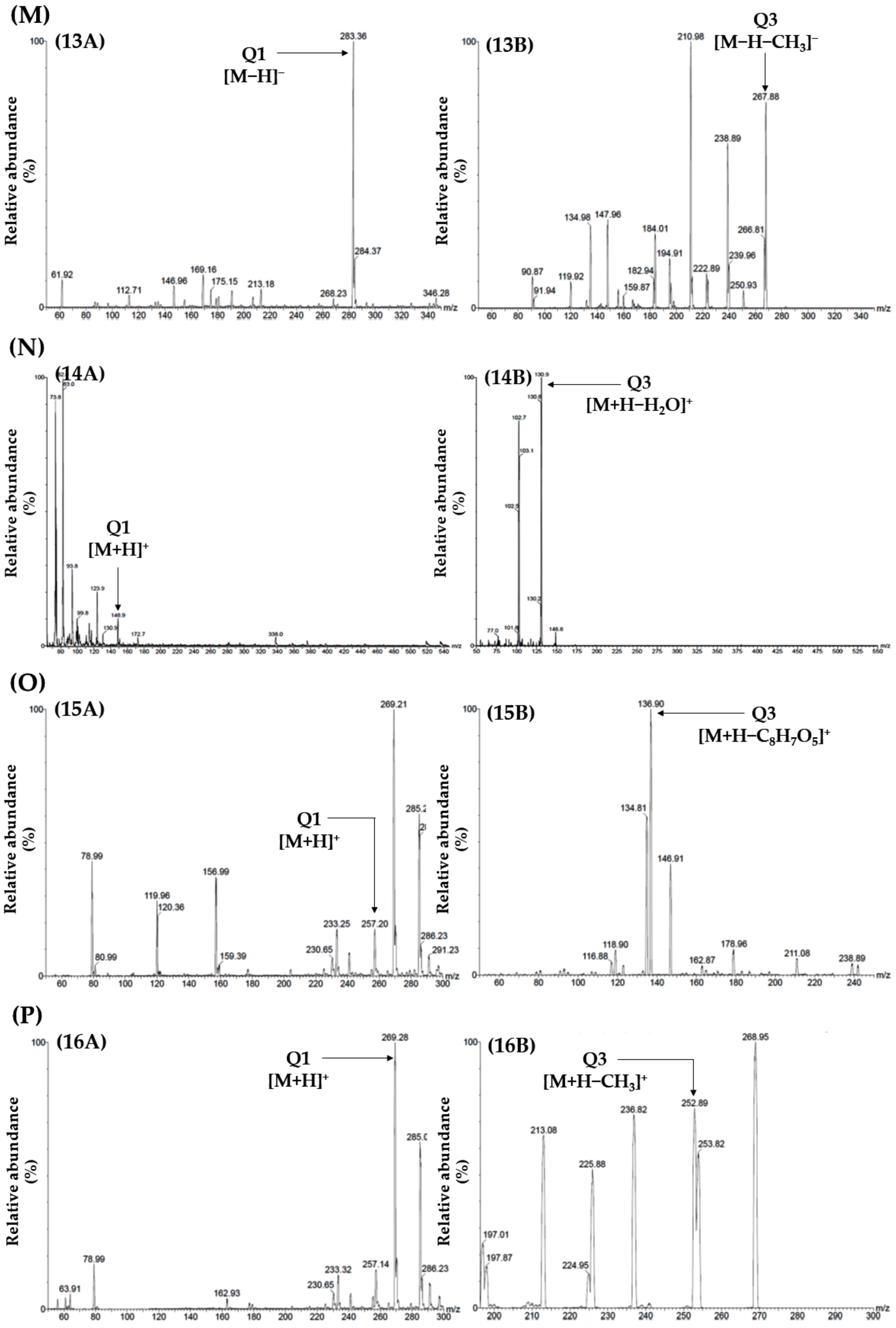
(M)
(13A)
Q1
[M−H]⁻
283.36
284.37
61.92
112.71
146.96
169.16
175.15
213.18
268.23
346.28
(13B)
Q3
[M−H−CH3]⁻
210.98
267.88
238.89
134.98
147.96
184.01
194.91
266.81
239.96
182.94
159.87
222.89
250.93
90.87
91.94
119.92
(N)
(14A)
Q1
[M+H]⁺
(14B)
Q3
[M+H−H2O]⁺
(O)
(15A)
Q1
[M+H]⁺
269.21
78.99
119.96
120.36
156.99
233.25
257.20
230.65
286.23
291.23
80.99
159.39
(15B)
Q3
[M+H−C8H7O5]⁺
136.90
134.81
146.91
118.90
116.88
178.96
162.87
211.08
238.89
(P)
(16A)
Q1
[M+H]⁺
269.28
78.99
63.91
162.93
233.32
257.14
230.65
286.23
(16B)
Q3
[M+H−CH3]⁺
268.95
252.89
253.82
236.82
213.08
225.88
224.95
197.01
197.87
Relative abundance
(%)
m/z

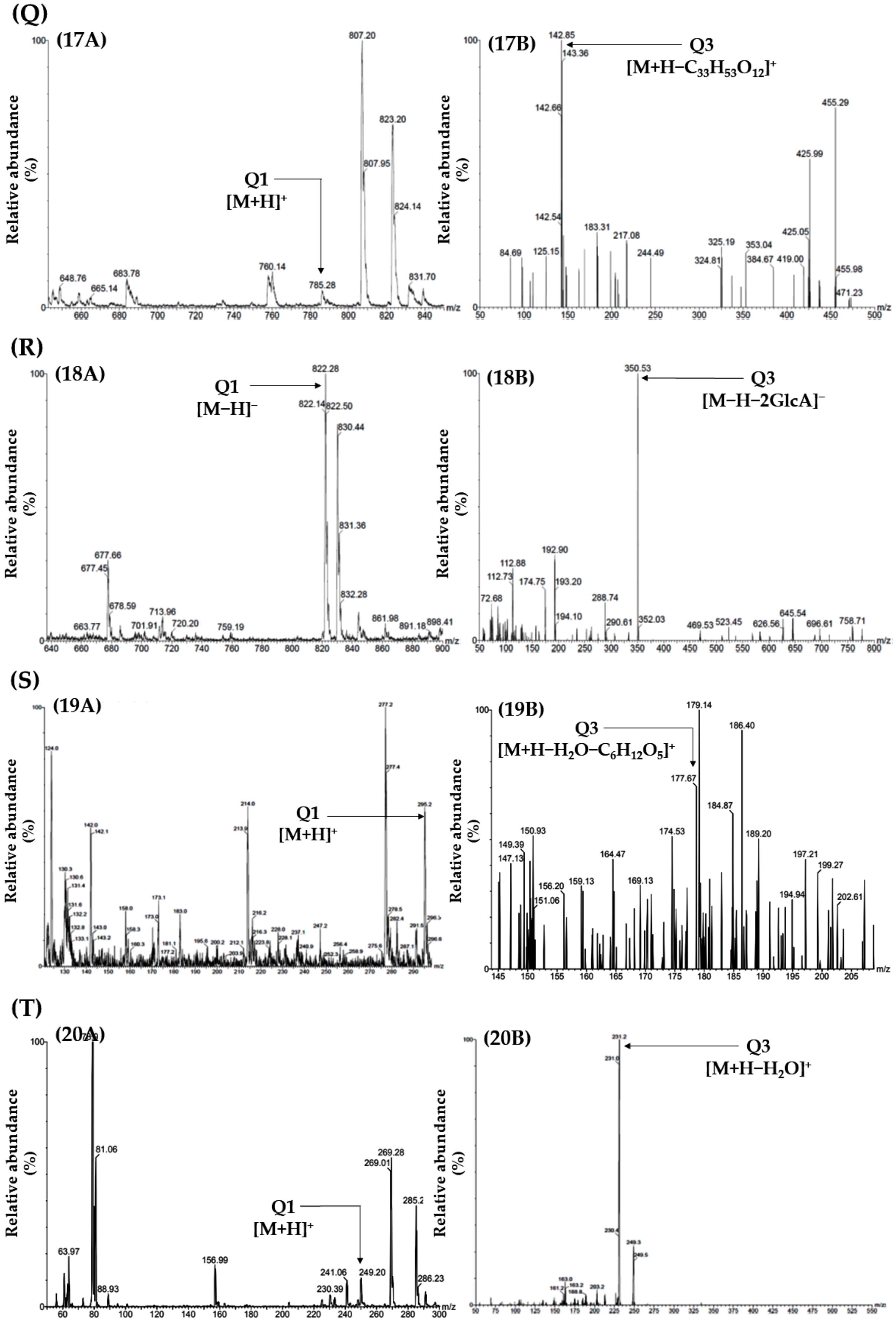
(Q)
(17A)
Q1
[M+H]+
Relative abundance (%)
807.20
823.20
807.95
824.14
760.14
785.28
831.70
648.76
683.78
665.14
m/z
(17B)
Q3
[M+H−C33H53O12]+
142.85
143.36
142.66
142.54
455.29
425.99
425.05
183.31
217.08
244.49
125.15
84.69
325.19
324.81
353.04
384.67
419.00
455.98
471.23
(R)
(18A)
Q1
[M−H]−
822.28
822.14
822.50
830.44
831.36
832.28
677.66
677.45
678.59
713.96
720.20
701.91
663.77
759.19
861.98
891.18
898.41
(18B)
Q3
[M−H−2GlcA]−
350.53
192.90
193.20
194.10
112.88
112.73
174.75
72.68
288.74
290.61
352.03
469.53
523.45
626.56
645.54
696.61
758.71
(S)
(19A)
Q1
[M+H]+
(19B)
Q3
[M+H−H2O−C6H12O5]+
179.14
186.40
177.67
184.87
189.20
174.53
150.93
149.39
147.13
164.47
169.13
156.20
159.13
151.06
197.21
199.27
194.94
202.61
(T)
(20A)
Q1
[M+H]+
81.06
63.97
88.93
156.99
269.28
269.01
285.2
241.06
249.20
230.39
286.23
(20B)
Q3
[M+H−H2O]+

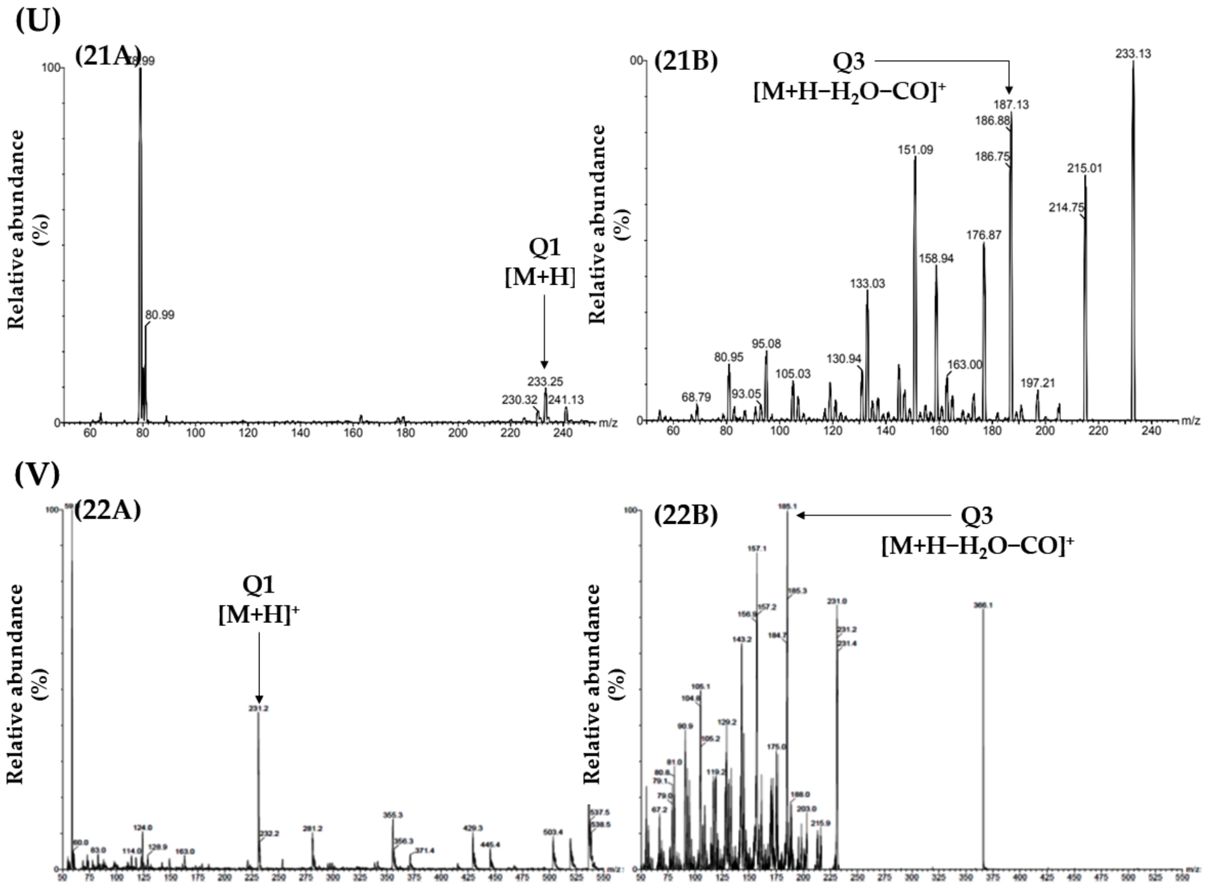

Figure S1. MRM mass spectra of the 22 marker compounds: Precursor ion (Q1, **Left-A**) and product ion (Q3, **Right-B**) peaks. Sinomenine (SIN, **A**), magnoflorine (MAG, **B**), rutin (RUT, **C**), liquiritin apioside (LIQA, **D**), calycosin-7-*O*-glucoside (CALG, **E**), liquiritin (LIQ, **F**), fangchinoline (FAN, **G**), tetrandrine (TET, **H**), isoliquiritin apioside (ILIQA, **I**), isoliquiritin (ILIQ, **J**), ononin (ONO, **K**), liquiritigenin (LIQG, **L**), calycosin (CAL, **M**), cinnamic acid (CINA, **N**), isoliquiritigenin (ILIQG, **O**), formononetin (FOR, **P**), astragaloside IV (AST IV, **Q**), glycyrrhizin (GLY, **R**), 6-gingerol (GIN, **S**), atractylenolide III (ATR III, **T**), atractylenolide II (ATR II, **U**), and atractylenolide I (ATR I, **V**).

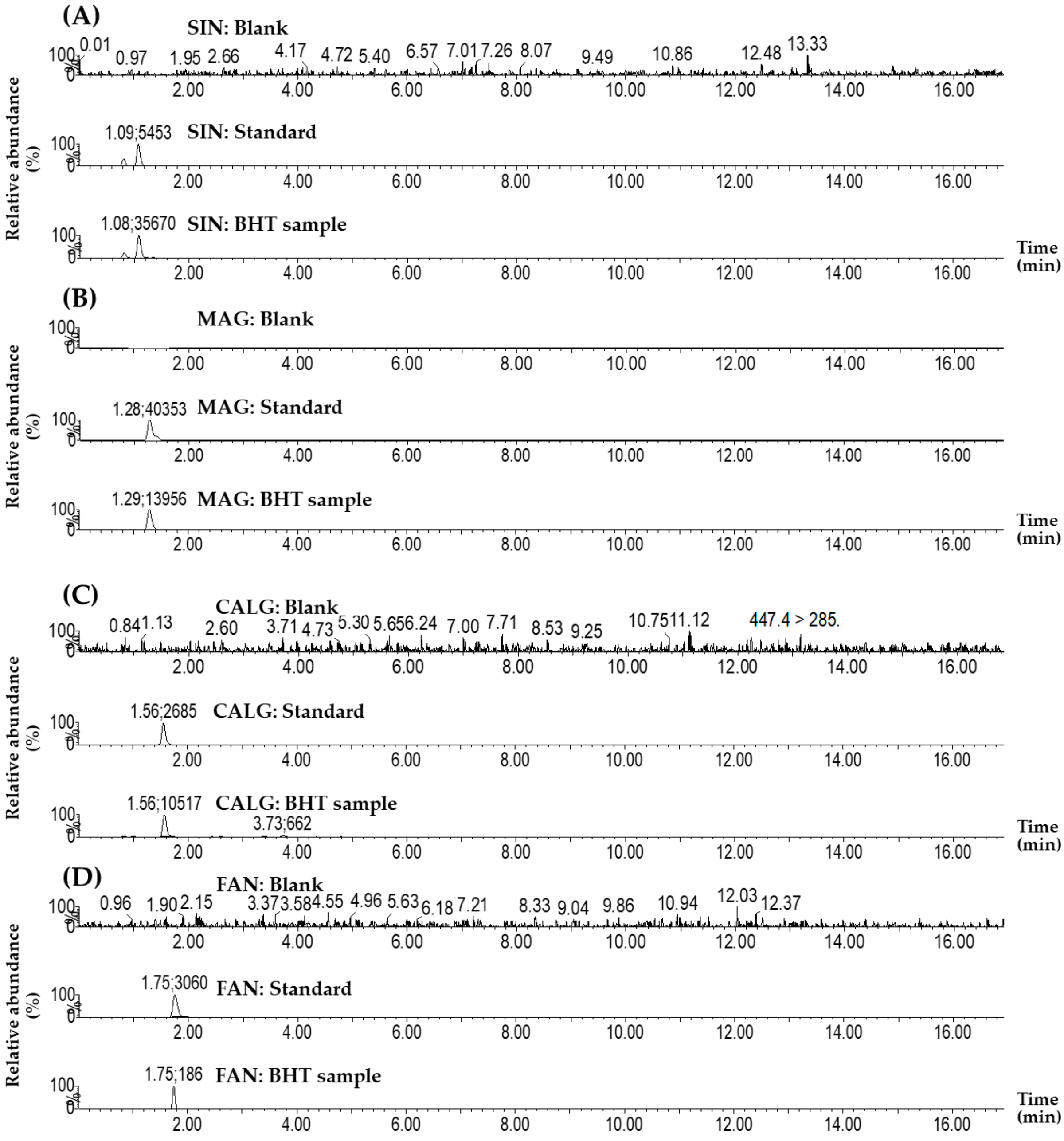

(A)
SIN: Blank
SIN: Standard
1.09;5453
SIN: BHT sample
1.08;35670
(B)
MAG: Blank
MAG: Standard
1.28;40353
MAG: BHT sample
1.29;13956
(C)
CALG: Blank
447.4 > 285.
CALG: Standard
1.56;2685
CALG: BHT sample
1.56;10517
3.73;662
(D)
FAN: Blank
FAN: Standard
1.75;3060
FAN: BHT sample
1.75;186
Relative abundance (%)
Time (min)

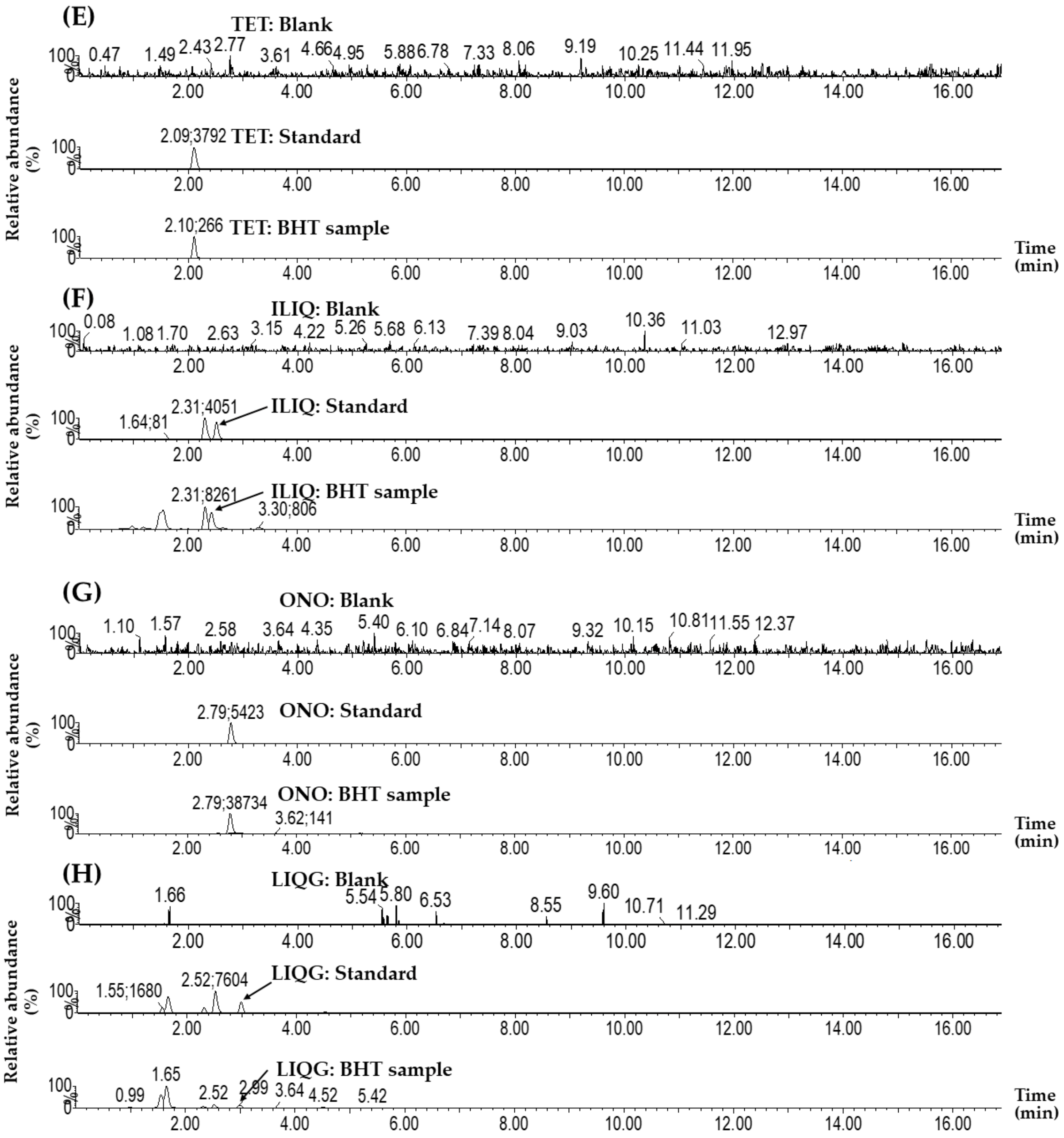

(E)
TET: Blank
TET: Standard
2.09;3792
TET: BHT sample
2.10;266
(F)
ILIQ: Blank
ILIQ: Standard
2.31;4051
1.64;81
ILIQ: BHT sample
2.31;8261
3.30;806
(G)
ONO: Blank
ONO: Standard
2.79;5423
ONO: BHT sample
2.79;38734
3.62;141
(H)
LIQG: Blank
LIQG: Standard
1.55;1680
2.52;7604
LIQG: BHT sample
Relative abundance (%)
Time (min)

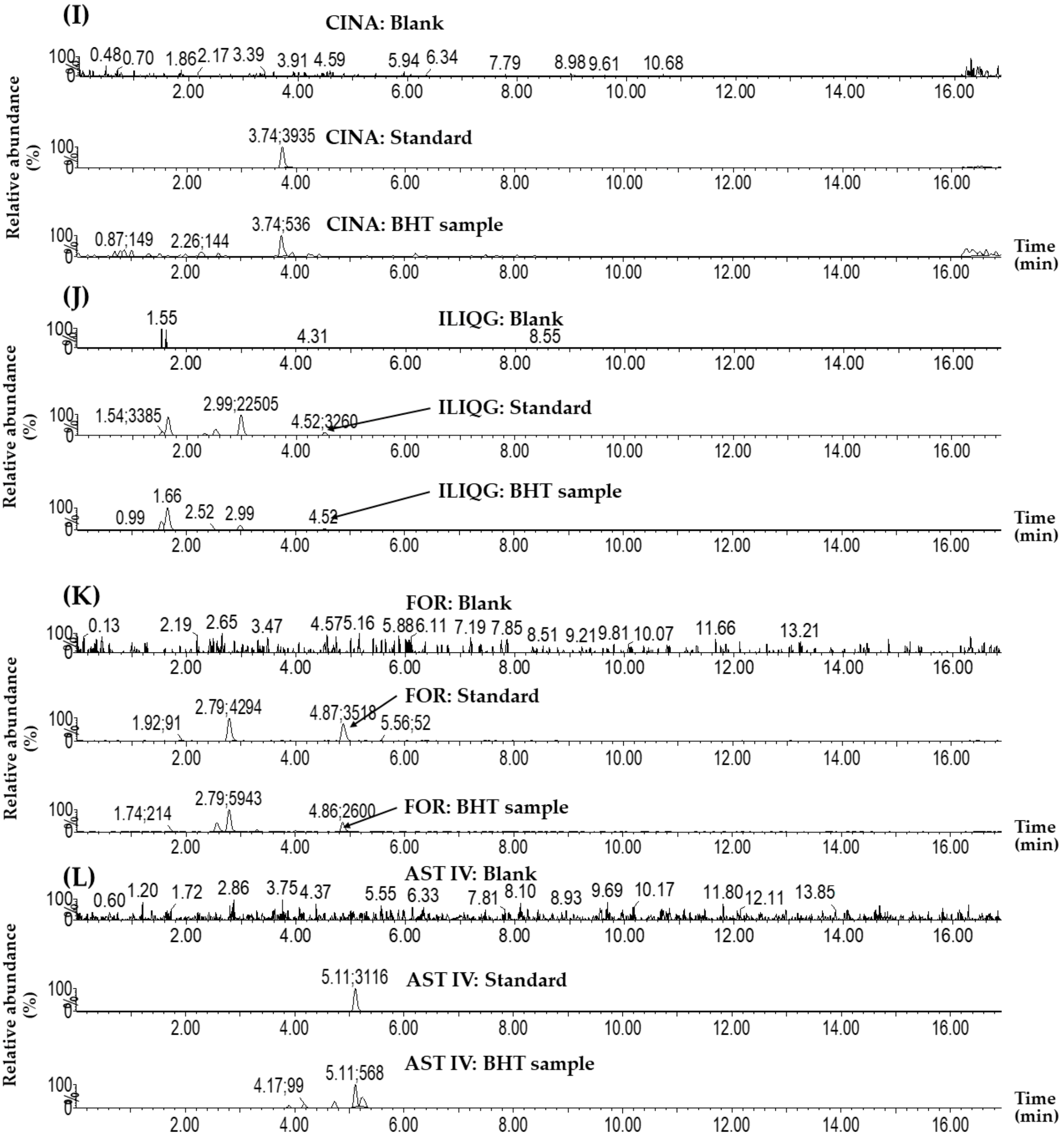
(I)
CINA: Blank
0.48 0.70 1.86 2.17 3.39 3.91 4.59 5.94 6.34 7.79 8.98 9.61 10.68
CINA: Standard
3.74;3935
CINA: BHT sample
0.87;149 2.26;144 3.74;536
(J)
ILIQG: Blank
1.55 4.31 8.55
ILIQG: Standard
1.54;3385 2.99;22505 4.52;3260
ILIQG: BHT sample
0.99 1.66 2.52 2.99 4.52
(K)
FOR: Blank
0.13 2.19 2.65 3.47 4.57 5.16 5.88 6.11 7.19 7.85 8.51 9.21 9.81 10.07 11.66 13.21
FOR: Standard
1.92;91 2.79;4294 4.87;3518 5.56;52
FOR: BHT sample
1.74;214 2.79;5943 4.86;2600
(L)
AST IV: Blank
0.60 1.20 1.72 2.86 3.75 4.37 5.55 6.33 7.81 8.10 8.93 9.69 10.17 11.80 12.11 13.85
AST IV: Standard
5.11;3116
AST IV: BHT sample
4.17;99 5.11;568
Relative abundance (%)
100
0
2.00 4.00 6.00 8.00 10.00 12.00 14.00 16.00
Time (min)

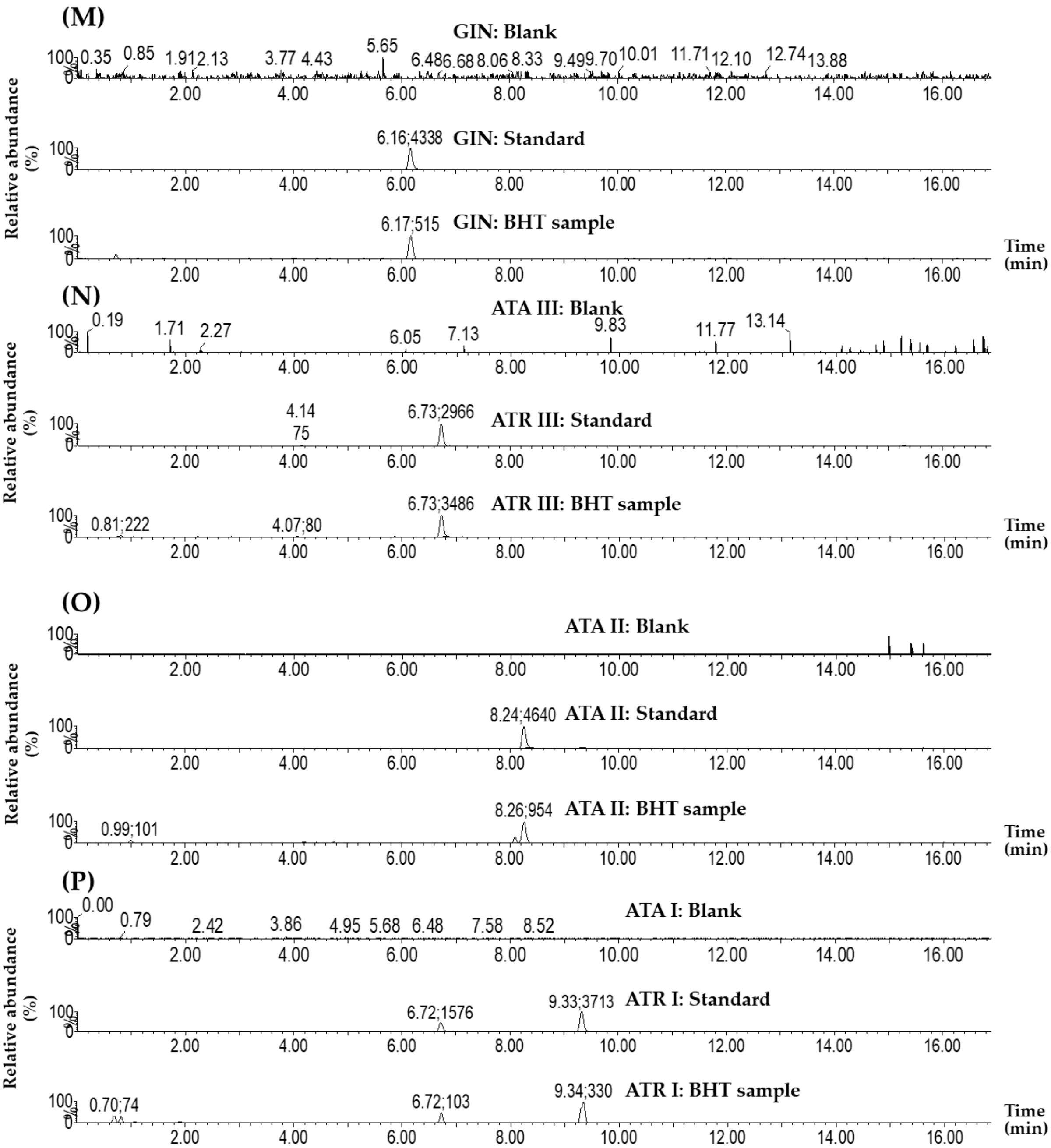
(M)
GIN: Blank
GIN: Standard
6.16;4338
GIN: BHT sample
6.17;515
(N)
ATA III: Blank
ATR III: Standard
6.73;2966
ATR III: BHT sample
6.73;3486
(O)
ATA II: Blank
ATA II: Standard
8.24;4640
ATA II: BHT sample
8.26;954
(P)
ATA I: Blank
ATR I: Standard
6.72;1576
9.33;3713
ATR I: BHT sample
6.72;103
9.34;330
Relative abundance (%)
Time (min)

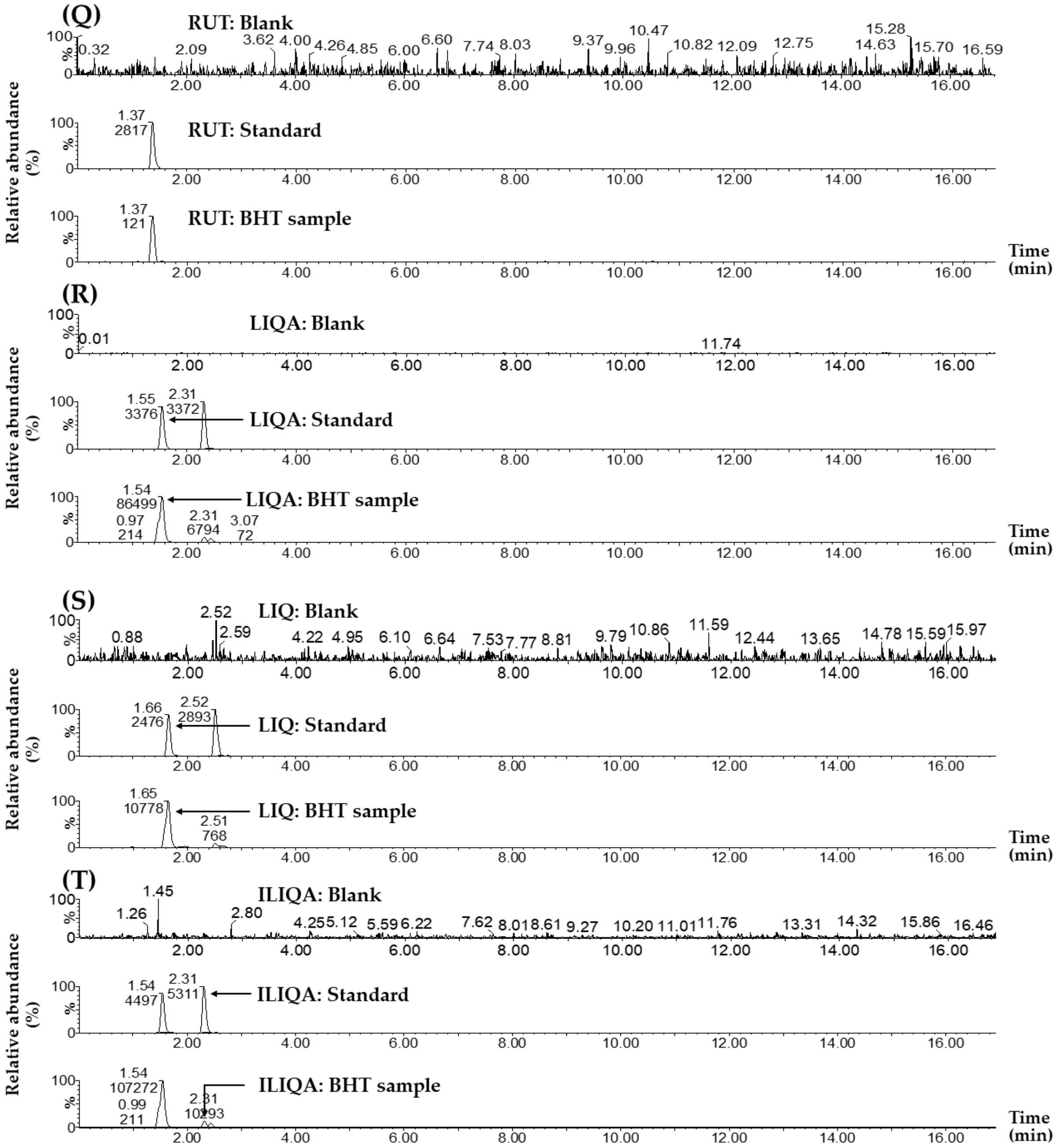
(Q)
RUT: Blank
RUT: Standard
RUT: BHT sample
(R)
LIQA: Blank
LIQA: Standard
LIQA: BHT sample
(S)
LIQ: Blank
LIQ: Standard
LIQ: BHT sample
(T)
ILIQA: Blank
ILIQA: Standard
ILIQA: BHT sample
Relative abundance (%)
Time (min)
1.37
2817
1.37
121
1.55
3376
2.31
3372
1.54
86499
0.97
214
2.31
6794
3.07
72
1.66
2476
2.52
2893
1.65
10778
2.51
768
1.54
4497
2.31
5311
1.54
107272
0.99
211

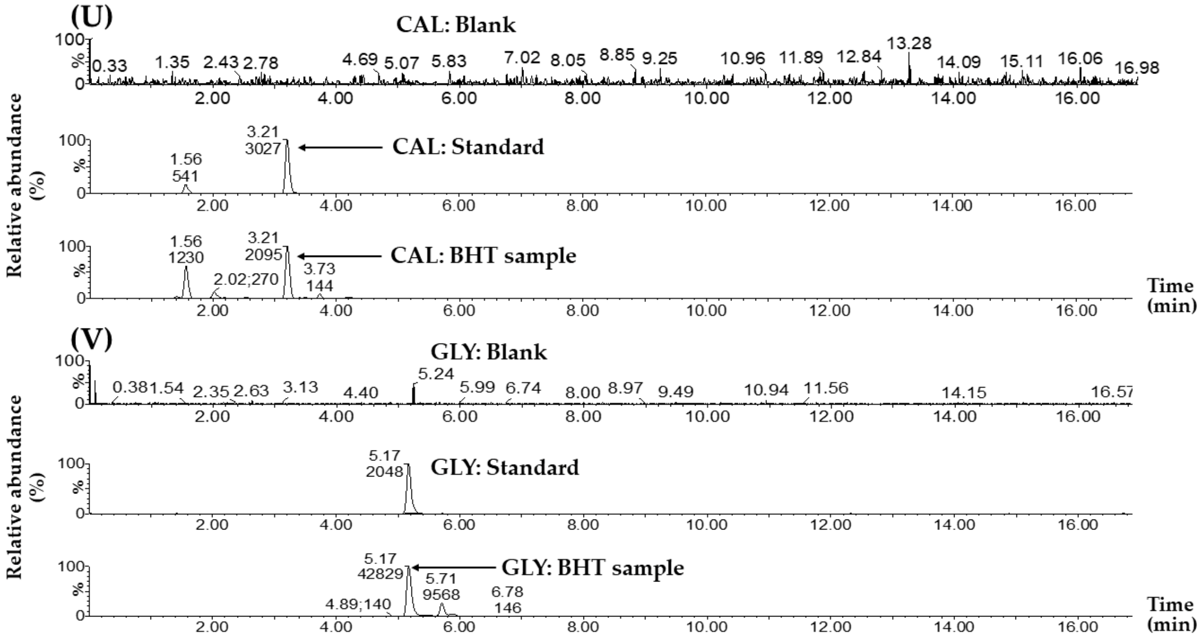

Figure S2. Extracted ion chromatograms of blanks, standard marker compounds, and BHT samples for selectivity evaluation by UPLC–MS/MS using MRM in positive (**A**–**P**) and negative (**Q**–**V**) ion modes. SIN (**A**), MAG (**B**), CALG (**C**), FAN (**D**), TET (**E**), ILIQ (**F**), ONO (**G**), LIQG (**H**), CINA (**I**), ILIQG (**J**), FOR (**K**), AST IV (**L**), GIN (**M**), ATR III (**N**), ATR II (**O**), ATR I (**P**), RUT (**Q**), LIQA (**R**), LIQ (**S**), ILIQA (**T**), CAL (**U**), and GLY (**V**).

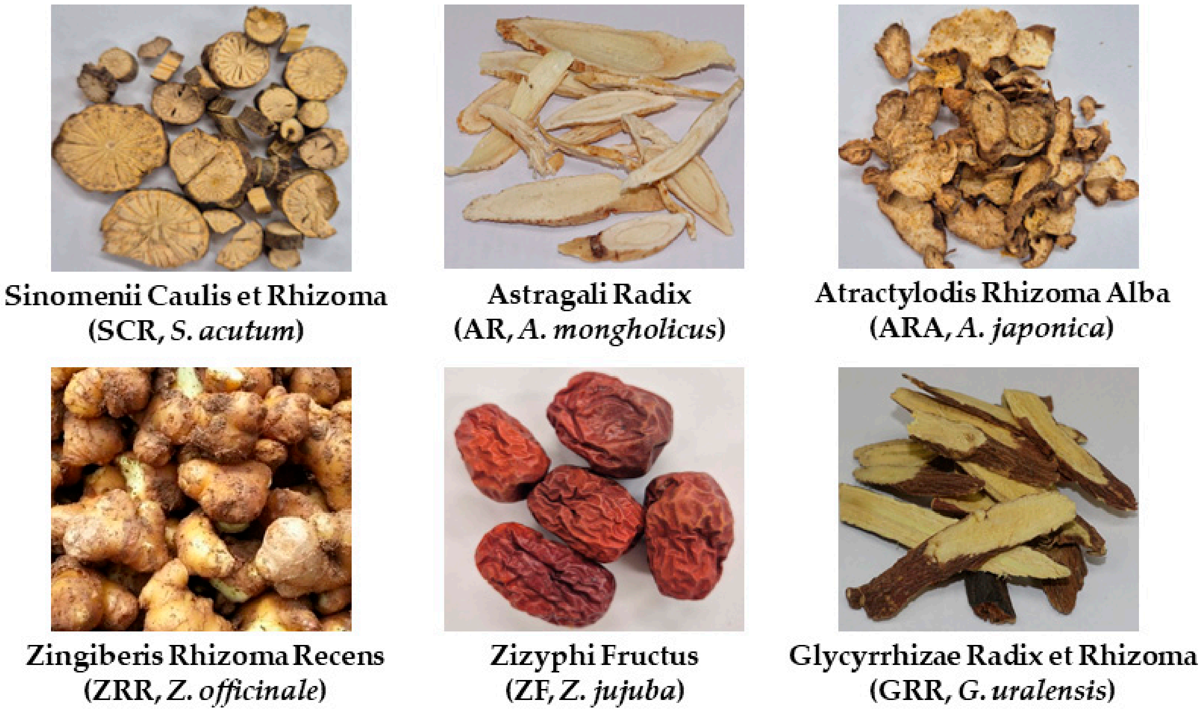

Figure S3. Six constituent herbal medicines of BHT

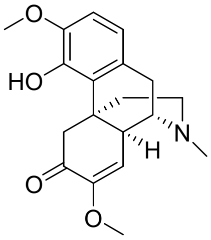

Sinomenine

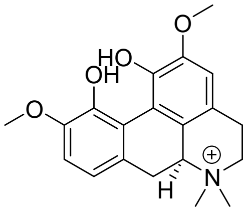

Magnoflorine

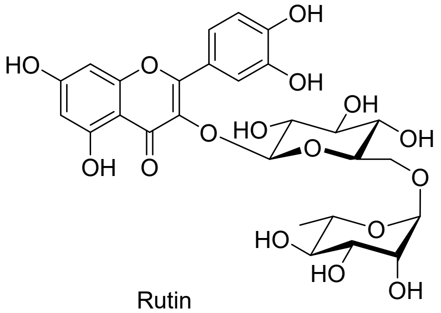

Rutin

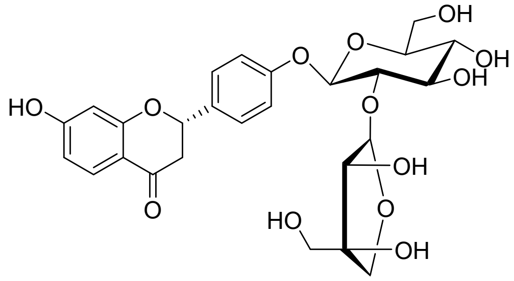

Liquiritin apioside

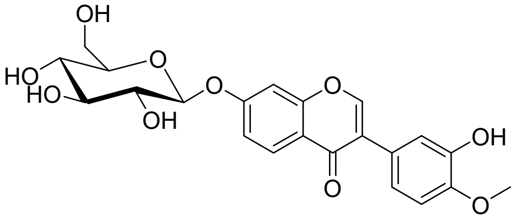

Calycosin-7-*O*-glucoside

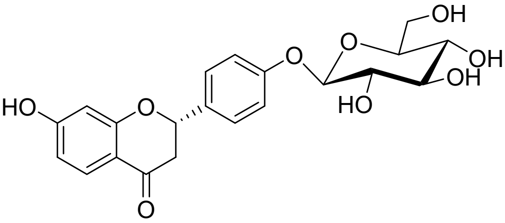

Liquiritin

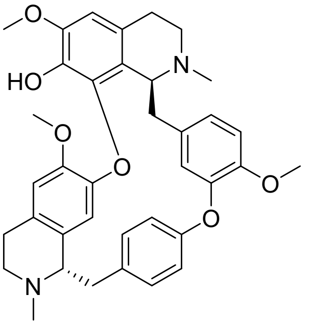

Fangchinoline

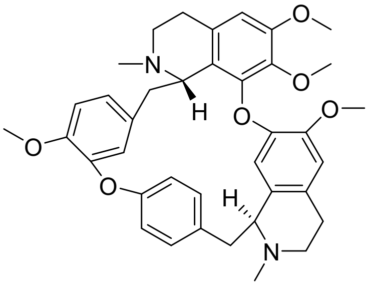

Tetrandrine

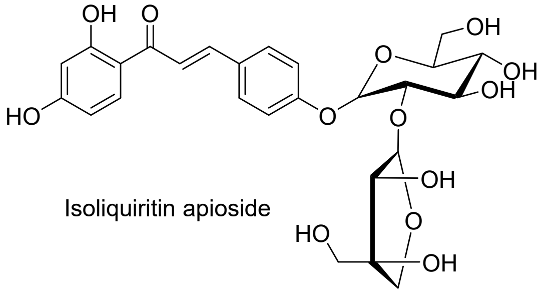

Isoliquiritin apioside

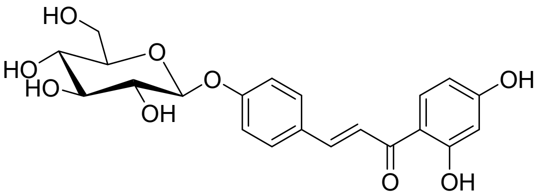

Isoliquiritin

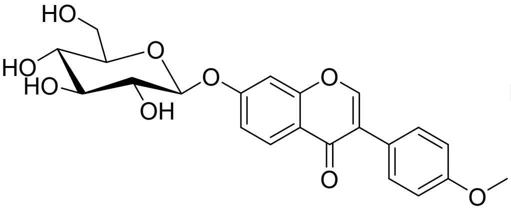

Ononin

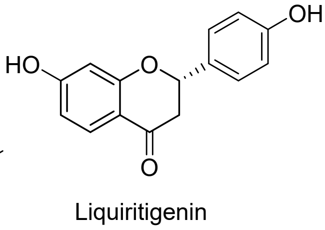

Liquiritigenin

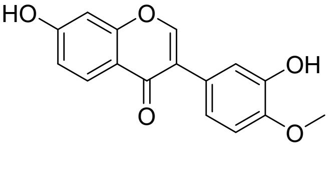

Calycosin

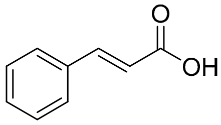

Cinnamic acid

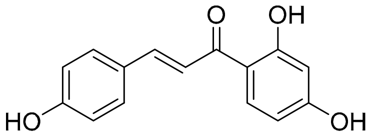

Isoliquiritigenin

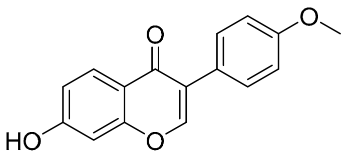

Formononetin

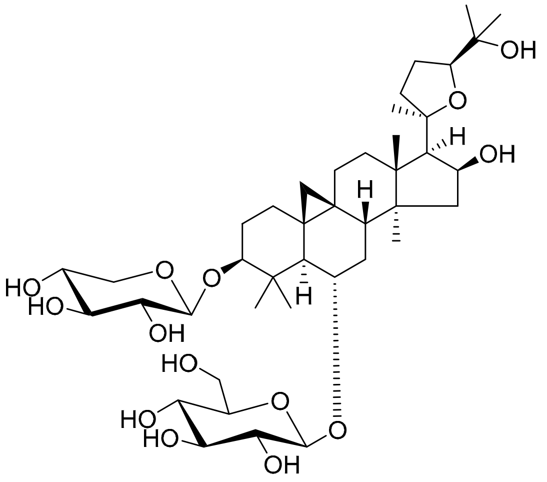

Astragaloside IV

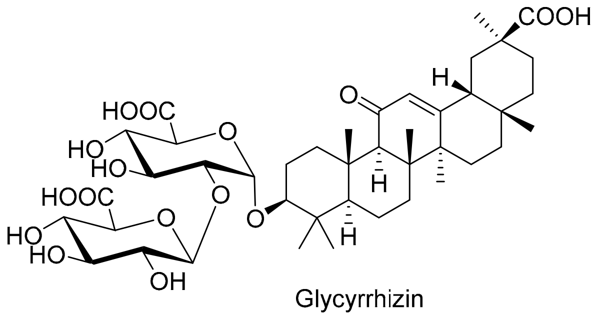

Glycyrrhizin

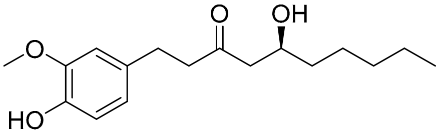

6-Gingerol

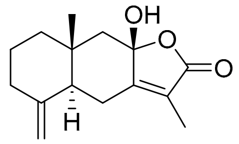

Atractylenolide III

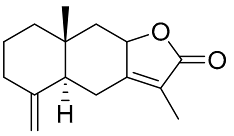

Atractylenolide II

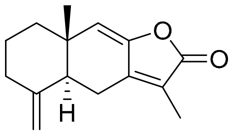

Atractylenolide I

Figure S4. Chemical structures of the 22 marker compounds for quality control of BHT.
